# S2k guidelines: diagnosis and treatment of varicose veins

**DOI:** 10.1007/s00105-022-04977-8

**Published:** 2022-04-19

**Authors:** F. Pannier, T. Noppeney, J. Alm, F. X. Breu, G. Bruning, I. Flessenkämper, H. Gerlach, K. Hartmann, B. Kahle, H. Kluess, E. Mendoza, D. Mühlberger, A. Mumme, H. Nüllen, K. Rass, S. Reich-Schupke, D. Stenger, M. Stücker, C. G. Schmedt, T. Schwarz, J. Tesmann, J. Teßarek, S. Werth, E. Valesky

**Affiliations:** Praxis für Dermatologie und Phlebologie, Helmholtzstr. 4–6, 53123 Bonn, Germany

## 1 Introduction

These guidelines deal with the diagnosis and treatment of subcutaneous varicose veins and intrafascial varicose veins; Association of the Scientific Medical Societies in Germany (AWMF) register number 037-018.

## 2 Abstract

### 2.1 Participating professional associations and organisations

These guidelines for the diagnosis and treatment of varicose veins were prepared under the guidance of the Deutsche Gesellschaft für Phlebologie e. V. (DGP) in cooperation with the Deutsche Gesellschaft für Gefäßchirurgie und Gefäßmedizin—Gesellschaft für operative, endovaskuläre und präventive Gefäßmedizin e. V. (DGG), the Deutschen Gesellschaft für Angiologie, Gesellschaft für Gefäßmedizin e. V. (DGA), the Deutsche Dermatologischen Gesellschaft (DDG), the Deutsche Gesellschaft für Dermatochirurgie e. V. (DGDC), the Berufsverband der Phlebologen e. V. (BVP), and the Arbeitsgemeinschaft der niedergelassenen Gefäßchirurgen Deutschlands e. V. (ANG). This updated 2018/2019 version is based on the guidelines agreed and drafted by the same associations in 2004 and 2009, and it was adopted by the Boards of the participating professional associations on 30 April 2019.

### 2.2 Development stage of the guidelines

These guidelines are based on a structured consensus process, drawing on published data to create consensus-based guidelines at development stage S2k.

### 2.3 Delegates of the professional associations

See: https://www.awmf.org/uploads/tx_szleitlinien/037-018l_S2k_Varikose_Diagnostik-Therapie_2019-07.pdf.

### 2.4 Selected literature

The recommendations are based on the same publications used in previous versions and a systematic literature review carried out on 21 July 2016 in the German Institute for Vascular Public Health Research (DIGG). The review included randomised studies, meta-analyses, and controlled studies. The literature search was carried out in the Medline and PubMed databases with the following search fields in German and/or English: sclerotherapy, endovenous thermal ablation, mechanochemical ablation, cyanoacrylate glue, surgical procedures (stripping), and diagnosis, prognosis, and postoperative care of varicose veins. A manual search was carried out for later publications up to December 2018.

### 2.5 Recommendations

The strengths of the consensus-based recommendations are based on the recommendations of the AWMF. The following levels are used:

- Shall/shall not: strong recommendation
- Should/should not: recommendation
- Can be considered/can be omitted: open recommendation

## 3 Method

See guideline report: https://www.awmf.org/uploads/tx_szleitlinien/037-018l_S2k_Varikose_Diagnostik-Therapie_2019-07.pdf.

## 4 General

### 4.1 Classification of the varicose vein

Varicose vein disease (varicose vein disease, primary varicose vein) is a degenerative disease of the vein wall in the superficial vein system of the legs in which, under the influence of a range of factors (e.g., pregnancy, orthostatic stress), more or less pronounced and severe varicose veins (varices) develop over the course of the patient’s life [1]. A varicose vein is a lifelong, progressive disease that can have a decisive negative impact on the patient’s quality of life. Nevertheless, a primary varicose vein is not in itself a life-changing disease. This fact shall always be borne in mind in all decisions taken with respect to varicose vein disease.

In the presence of a primary varicose vein, it is essential to distinguish the (primary) varices from those that may develop as a result of obliterate processes in the deep vein system as epifascial collateral veins (secondary varices).

The following basic types of varicose veins can be distinguished, based on topographical and/or morphological criteria:

- Varicose saphenous veins, including varicose accessory saphenous veins
- Varicose tributary veins
- Varicose perforator veins
- Pelvic varices
- Reticular varices
- Spider veins

Veins should be indicated anatomically and topographically using the terms defined in the nomenclature developed in a transatlantic consensus document [2]. According to Hach, haemodynamically significant saphenous vein incompetence can be classified into different degrees (classes) in the refluxing segment [3]; however, these classes do not cover every variation of varicose veins. In the case of incompetence in the junction region of a saphenous vein, the incompetence class is defined by the length of the refluxing segment to the distal reflux point.

Other forms of haemodynamically significant varicose veins exist [4], such as the following:

- Incomplete saphenous varicose vein (proximal reflux source in a perforator vein or in another region of the saphenous vein)
- Ascending varicose vein without primary incompetence in the junction region
- Isolated varicose tributary and perforator vein
- Special forms of varicose veins, e.g., pudendal, gluteal, and pelvic varicose veins [5, 6]

The region of the saphenofemoral junction presents a multitude of variants, with or without terminal valve incompetence and with or without incompetence of the preterminal valve [7]. These result in different forms of saphenofemoral incompetence. Similar behaviour is found in the region of the saphenopopliteal junction.

### 4.2 Natural development of the varicose vein

A large number of epidemiological studies have shown that primary varicose veins are a very frequent disease [8–10]. In the Bonn Vein Study of 2003, one man in six and one woman in five presented chronic venous insufficiency (CVI); however, severe expressions of the disease had diminished in comparison with older epidemiological studies. In this study, 12.4% of men and 15.8% of women presented varicose veins without signs of CVI, and 11.6% of men and 14.9% of women presented venous oedema. Advanced CVI (CEAP classification: C4–C6) was found in 3.8% of men and 3.4% of women [11].

Basic risk factors for varicose veins are advanced age, female sex, pregnancies, and positive family history [12, 13].

Primary varicose vein disease may appear in childhood, and its prevalence increases with age [12, 14]. A genetic disposition is assumed for primary varicose veins [12].

The varicose veins may appear with or without symptoms (C2) or with oedema and/or skin alterations in the context of CVI (C3–C6) [15, 16]. If it is not treated, a medically significant varicose vein, particularly an incompetent saphenous or perforator vein, frequently leads to complications (chronic oedema, trophic skin alterations, venous leg ulcer, deep vein incompetence, varicophlebitis) [13, 17, 18]. An important pathogenetic factor is disturbance of venous haemodynamics, with the development of ambulatory venous hypertension. This leads to increased risk of deep leg vein thrombosis, especially with the simultaneous presence of superficial vein thrombosis [19]. In the Basel study, it was found that individuals with serious or painful varicose veins suffered between nine and 20 times more complications of the venous system, depending on the degree of severity, than individuals of the same age who were free of varicose vein disease [20]. Epidemiological and prospective studies have also confirmed that the presence of a symptomatic varicose vein causes quality of life to deteriorate [21, 22].

Varicose vein disease is progressive over the course of life. In the Edinburgh Vein Study, after 13.4 years, 57.8% of patients with a varicose saphenous vein or chronic venous incompetence presented progression (4.3%/year) [18]. Labropoulos showed that after 19 months, 14.7% of varicose vein patients presented a longer refluxing segment, and 11.2% a progression of the clinical alterations [23]. Engelhorn showed that young patients with early stages of varicose vein disease often present ascending progression of the varicose vein [24]. In a population of 304 patients on the waiting list for an operation on a varicose saphenous vein in England, after 4 years Brewster found progression of the disease in 64% of cases: 5.2% developed a superficial vein thrombus, 22% developed skin alterations, and 12% developed a venous leg ulcer [25]. In the Bonn vein study, after 6.7 years, 19.8% of the patients with varicose nonsaphenous veins and 31.8% of patients with varicose saphenous veins, all in CEAP stage C2, developed progression to CVI [13].

### 4.3 Indications for varicose vein treatment and referral to a specialist

The objective of varicose vein treatment consists of

- normalisation or improvement of vein haemodynamics,
- improvement or elimination of congestion pain (heaviness, tension, heat, pain) and/or persistent oedema,
- healing, or reduction of the recurrence rate, of venous ulcers and other forms of trophic disturbances, and
- prevention of further complications, e.g., superficial vein thrombosis, deep vein incompetence, arthrogenic congestion syndrome, variceal bleeding [26, 27].

Varicose veins may be asymptomatic or symptomatic (painful symptoms). They may lead to the development of oedema and/or skin alterations up to and including venous ulcers. Furthermore, multiple complications may appear, such as superficial vein thrombosis, vein inflammation, or variceal bleeding.

#### Recommendation 1

In the presence of a symptomatic or significant varicose vein, a special examination should be carried out to plan further procedures. To this end, the patient should be referred to a vein specialist who has sufficient knowledge about the full spectrum of diagnosis, including duplex ultrasound, and the range of possible treatments and/or can make appropriate recommendations.

#### Recommendation 2

Any patient with variceal bleeding, superficial vein thrombosis, varices during pregnancy, or a venous leg ulcer shall be referred to a vein specialist for further treatment planning.

#### Recommendation 3

The treatment of a varicose vein and its complications can be conservative or invasive. Choice of treatment is dictated by the will of the patient, the severity and location of the pathological alterations to the superficial and deep vein systems, and the patient’s general state of health.

#### Recommendation 4

For patients with varicose veins and signs of CVI (venous oedema to venous leg ulcer), a haemodynamically effective treatment shall be sought.

#### Recommendation 5

In the presence of varicose vein complications (variceal bleeding, superficial vein thrombosis, venous leg ulcer), prompt, appropriate treatment shall be sought.

### 4.4 Classification and diagnosis

Characterisation of the varicose vein and its effects shall/should be developed jointly with diagnosis of a state or class using a recognised classification system. The CEAP classification for the description of chronic vein diseases [28] has been internationally established. The clinical classification according to CEAP is shown in Table 1.

**Table 1:** Clinical classification (*C*) according to CEAP

Class	Clinical signs
C_0_	No visible or palpable signs of venous incompetence
C_1_	Spider veins and/or reticular varices
C_2_	Varicose veins
C_3_	Oedema
C_4a_	Pigmentation, eczema
C_4b_	Atrophie blanche, dermatoliposclerosis
C_5_	Cured venous leg ulcer
C_6_	Active venous leg ulcer

Any C class can present without or with subjective symptoms such as pain or sensations of heaviness, tension, or swelling. In symptomatic patients, a subscript *s* is added (e.g., C_2s_ = symptomatic varicose vein). Chronic venous incompetence (CVI) is defined as C class C_3_–C_6_.

In addition, the CEAP classification can be complemented with aetiological (E), anatomical (A), and pathophysiological (P) criteria as needed. The CEAP classification does not indicate the severity of the varicose vein. The intention of the initiators and developers of the CEAP classification was that it should provide a clinical classification valid for all types of chronic venous diseases.

#### Recommendation 6

The CEAP classification should be used for classifying a varicose vein.

Apart from the CEAP system, other validated classification systems exist, used principally in the context of scientific studies. The degree of severity of vein disease can be indicated with the Venous Clinical Severity Score (VCSS) [29], among other systems. The impacts of vein disease on quality of life can be measured and described, for example, with the following score systems: the Venous Insufficiency Epidemiologic and Economic Study of Quality of Life (VEINES-QOL/Sym) [30], the Chronic Venous Insufficiency Qualify of Life Questionnaire (CIVIQ) [31], the Aberdeen Varicose Vein Questionnaire (AVVQ) [32], and the Freiburger Questionnaire of Quality of Life (FLQA) [33]. Shortened versions of many of these systems have been described for use in clinical practice.

#### Recommendation 7

Validated clinical scores and quality-of-life scores can be used to indicate the severity of a varicose vein.

### 4.5 Patients’ expectations, and explanations given to the patient

The patients’ expectations of planned treatments can be very variable, ranging from cosmetic aspirations through symptom relief and healing of complications or an ulcer to halting progression of the disease. Many patients are motivated by anxiety or fear about the invasiveness and the side effects of treatment measures [34].

Thorough, comprehensive explanation of the many methods available, the advantages and disadvantages of a treatment, possible complications and prospects for success, etc., as well as complete, traceable documentation, are indispensable for the legal validity of a declaration of consent. In Germany, the entire complex of explanation, consent, and documentation has been governed by a special law since 2013 (Law for the Improvement of Patients’ Rights, §§ 630c, d, e, f BGB), known as the Patients’ Rights Law.

#### Recommendation 8

The patient’s wishes shall be taken into account in the explanations and the selection of the treatment method. They form an important part of the treatment decision.

#### Recommendation 9

The explanatory conversation shall discuss the type, severity, possible complications, and prognosis of the clinical picture. The different treatment options shall be explained, with their advantages and potential risks.

#### Recommendation 10

Patients shall be informed of the principles and the effectiveness and side effects of the suggested treatment, and of the possibilities of coverage by health insurance.

#### Recommendation 11

The explanation should include the fact that the course of vein diseases is in principle progressive and that, as a rule, a long-term, conservative, symptom-oriented treatment shall be applied.

## 5 Diagnosis

This chapter does not describe the diagnostic procedures as such, since each is the subject of its own guideline. Here only the validity of the procedure is indicated, in conjunction with the treatment decision in the case of a varicose vein.

Before varicose vein treatment can be planned, the disease shall be recognised, with its severity and differential diagnosis to distinguish it from other diseases. Table 2 shows a classification of the objects of diagnosis.

**Table 2:** Objects of diagnosis of varicose veins

The objects of diagnosis of a varicose vein disease are as follows:
Discovery and classification of the haemodynamic disturbance (duplex ultrasound)
Classification of the medical significance (medical record, inspection, duplex ultrasound, light reflection rheography [LRR], venous occlusion plethysmography [VOP])
Differentiation of a primary from a secondary varicose vein (duplex ultrasound, occasionally other imaging techniques, LRR, VOP)
Discovery and classification of the deep vein system (duplex ultrasound, phlebography, other imaging techniques, LRR, VOP)
Follow-up for quality control after the intervention (medical record, inspection, duplex ultrasound, LRR, VOP)
Analysis of recurrence (cause, extent) (duplex ultrasound, inspection)

*Objects of diagnosis with accompanying trophic disturbances or skin alterations such as oedema, as well as pain:*

- Exclusion or confirmation and description of an oedema and clarification of the cause, particularly diagnosis to differentiate oedema caused by an internal ailment—lymphoedema, lipoedema, obesity oedema—from venous oedema (inspection, medical record, recommendation for further investigation)
- Differential diagnosis of skin alterations present, if any (medical record, inspection)
- Differential diagnosis of painful symptoms present, particularly for differential diagnostic clarification of orthopaedically caused symptoms (inspection, physical examination, medical record, consultation with a neurologist/orthopaedic specialist)

*The following are required before a treatment can be indicated:*

- Exclusion of accompanying peripheral arterial occlusion disease (medical record, inspection, Doppler [ABI], duplex ultrasound)
- Exclusion of acute thrombotic event (ultrasound if there is clinical suspicion)
- Exclusion of infection (medical record, inspection, laboratory test if necessary)

### Recommendation 12

Investigation of the patient’s medical record and clinical examination shall form the basis of the decision for a diagnosis on which further action is planned.

Treatment planning, particularly the choice of the best treatment that will minimise complications and recurrence, is possible only after haemodynamic analysis of the varicose vein. This includes recording and describing the refluxive segments of the saphenous veins and tributaries, the sources of reflux from the deep vein system, and the reentry points of the recirculating blood, as well as analysis of the flow patterns in the deep vein system. The decision of when to start further treatment in addition to compression is also subject to quantification of the haemodynamic effects of the disease, for example by PPG or VOP, particularly in combination with the necessary progression check-up with comparative control.

### Recommendation 13

An imaging technique shall be used in the context of standardised phlebological diagnosis. The first-choice method shall be duplex ultrasound. If necessary, vein function shall also be measured (e.g., PPG/LRR or VOP).

### Recommendation 15

Complete, traceable documentation of the findings shall be kept.

### Recommendation 16

Pathological findings from technical examinations should be tested for clinical significance.

The methods listed below are regarded as standard procedures in clarifying and evaluating vein disease. They are not to be considered as competing alternatives. On the contrary, the combination of their different diagnostic powers and their assessment of different functional and morphological criteria will enable the sum of the results to achieve maximum diagnostic accuracy and reliability.

### 5.1. Diagnostic instruments

#### 5.1.1 Medical record

Indication and recommendations for application

The patient’s medical record shall be consulted at the time of the first examination. It contains information on the patient’s prior history (how long he or she has suffered varicose veins and which symptoms are present, especially swellings, skin alterations, and itching) and which measures relieve the symptoms (e.g., compression). At the same time, the doctor can also discover the record of drugs prescribed and inquire about thrombosis in the patient’s own record and that of his or her family, as well as ulcers and other previous ailments.

Further enquiry will reveal how the symptoms have altered and how good the patient’s adherence to compression is.

This information will give indications about the severity of the symptoms and thus about a possible line of treatment. The most common symptoms with a varicose vein are a sensation of heaviness, swelling, itching, and occasional pain after standing or sitting for long periods [16].

Documentation

The facts from the patient’s medical history shall be extracted traceably from the documentary record to allow follow-up of the progression of the symptoms and to note changes in the symptoms after the application of treatment.

##### Recommendation 17

In cases of venous symptoms, the medical record shall be the basis for diagnosis and differential diagnosis.

#### 5.1.2 Clinical examination

Indication

The clinical examination shall be the indispensable prerequisite for phlebological diagnosis. During the inspection, the doctor should look not only for visible varicose veins in the thigh and lower leg—especially the preferential areas in the lower leg—but also for oedema, hyperpigmentation, eczema, and (healed) ulcers, which may be related to chronic venous incompetence. In palpation, look out for stringy subcutaneous hardness, which may indicate acute or past superficial vein thrombosis. Larger areas of subcutaneous hardness may be an indication of (lipo)dermatosclerosis.

Execution

In a mobile patient, the clinical examination should be carried out with the patient standing up.

Documentation recommendations

The clinical expression of a varicose vein should be documented using the CEAP classification [28, 35]. Further information to help assess the evolution of the condition, especially after therapeutic interventions, can be recorded using the Venous Clinical Severity Score (VCSS) [36].

##### Recommendation 18

The clinical examination shall be taken into the treatment decision consideration as appropriate, as it reflects the severity of the disease.

#### 5.1.3 Global measurement techniques (photoplethysmography, also known as light reflection rheography, and venous occlusion plethysmography)

##### 5.1.3.1 Photoplethysmography (PPG).

Photoplethysmography (also called light reflection rheography, LRR) is described in a specific guideline [37].

Indication

Photoplethysmography or LRR can be used to control the evolution of the disease and can therefore be used on first contact with the patient and in subsequent controls, whether or not invasive treatment has been carried out.

If refilling time improves after application of a tourniquet to prevent reflux in the superficial vein system (e.g., above an incompetent perforator vein), this will enable the examining doctor to judge whether an invasive therapeutic measure in the superficial veins will produce a beneficial effect when there is a simultaneous pathology of the deep leg veins.

Results

Photoplethysmography correlates with C class (CEAP) and VCSS, two clinical severity scores, and the diameter of the great saphenous vein (GSV) [38, 39]. In study groups, the mean value has been found to improve after rehabilitation of the haemodynamic disturbance of the varicose vein [40–44].

No undesired effects are to be expected with this procedure. The interpretability is limited if the muscle pump effects evacuation of less than 3%. The procedure is sensitive to disturbances; false negatives may be recorded if the room temperature falls or if the patient has not rested before the examination.

##### Recommendation 19

Photoplethysmography (PPG, LRR) can be used to quantify venous function as a screening method and/or to control evolution.

##### Recommendation 20

An indication for invasive treatment shall not be based exclusively on PPG examination.

##### 5.1.3.2 Venous occlusion plethysmography (VOP).

Venous occlusion plethysmography (VOP) is a procedure for measuring pressure-dependent venous capacity, venous flow, and active volume evacuation [45], thus allowing conclusions to be drawn about the functioning of the deep leg veins.

##### Recommendation 21

Venous occlusion plethysmography can allow conclusions to be drawn about the functioning of the deep leg veins.

#### 5.1.4 Continuous wave (CW) Doppler ultrasound

Doppler ultrasound converts the blood flow in the vessels into an acoustic signal that can be displayed graphically. All blood flows within the cone of the Doppler beam are captured; it is not possible to distinguish between one vessel and another lying behind it.

Continuous wave (CW) Doppler ultrasound can be used in the initial screening if duplex is not available. Before invasive treatment, the varicose vein diagnosis should be confirmed with duplex ultrasound [46, 47].

Continuous wave Doppler ultrasound can also be used in the initial diagnosis of peripheral arterial occlusion disease [48].

This diagnosis is important for the treatment of varicose veins before compression treatment or the execution of surgery if peripheral arterial occlusion disease is suspected.

Please see the guidelines for the treatment of peripheral arterial occlusion disease for recommendations on how to measure occlusion pressure, including the ankle-brachial index (ABI).

##### Recommendation 22

An indication for invasive treatment shall not be based exclusively on CW Doppler ultrasound examination.

#### 5.1.5 Duplex ultrasound

Indications

Duplex ultrasound (DUS) is a noninvasive method of examination to detect the underlying haemodynamics of the varicose vein [46, 49]. It also provides information on pathologies of the deep vein system. Duplex ultrasound provides information on vein morphology, the anatomical classification of the pathological findings, and the diameter, occlusion, valve competence, and direction of flow in all three vein types. This information should be obtained for symptomatic varicose veins before advice is given on the need for treatment and its extent and type [46].

##### Recommendation 23

Duplex ultrasound shall be used as the basis for differentiated indications for the treatment of varicose veins. It should also be used for check-ups after invasive treatment of varicose veins.

##### Recommendation 24

Duplex ultrasound shall be used for parallel diagnosis during the execution of endovenous varicose vein treatment.

Duplex ultrasound is used to ascertain the cause of chronic venous incompetence in the initial diagnosis of a varicose vein.

Control of the evolution of the disease is recommended if there is clinically visible progress, as well as subsequently depending on the clinical findings.

After an intervention or operation, an early initial check-up is recommended, depending on the clinical course and the type of intervention. Further check-ups should be carried out after 1–3 months to document the early outcomes of the intervention and to detect early recurrence. Further controls will depend on the clinical evolution [46, 49, 50].

Recommendations for execution

Duplex ultrasound should be carried out with a linear probe at frequencies suitable for superficial areas. Information can be obtained in B scan, colour-coded duplex ultrasound and pulsed wave (PW) Doppler mode [49, 50].

##### Recommendation 25

Clinical examination of varicose veins and duplex ultrasound evaluation of the vein system should be carried out with the patient standing up.

Physical manoeuvres should be used, such as the Valsalva manoeuvre, or, preferably, manual compression of the calf in the standing patient, as well as dynamic manoeuvres [49, 51–53].

Duplex ultrasound should be documented by drawings or text, accompanied by diagnostic ultrasound images. Reflux shall be documented by an image of the flow curve along the time axis, and by the PW flow curve.

Results

The result of the examination is an understanding of the recirculation circuit, including the differentiated findings of the state of the deep vein system [54]. The particular anatomy of certain regions and the variant junctions of the small saphenous vein (SSV) in the popliteal fossa [55] or the variant courses of the GSV shall be shown traceably [54].

##### Recommendation 26

The proximal reflux source of the varicose vein shall be documented.

Differential examination of terminal and preterminal valves of the GSV allows different types of reflux to be identified [7, 56, 57]. Measuring the diameter of the GSV in the thigh (15 cm distal to the groin) can provide valuable information about the severity of the varicose vein [38, 39] and the risk of recurrence [58, 59].

Undesired effects

Duplex examination with dynamic manoeuvres presents no known risks. Extended examination of the standing patient and use of the Valsalva manoeuvre can lead to temporary vasovagal reactions up to and including syncope.

Limited interpretability

The interpretability of the examination depends not only on the experience of the examiner but also on the condition of the patient (e.g., bedridden, obese).

#### 5.1.6 Phlebography

Phlebography by vein X‑ray using contrast medium was the gold standard for vein diagnosis by imaging for decades. With the wide availability of duplex ultrasound, phlebography has disappeared from routine diagnosis.

Indication

Phlebography can be used as a complementary examination method if the duplex findings are unclear, or to exclude the suspicion of special conditions such as angiodysplasia, pudendal varicose vein, pelvic congestion, or doubts over collateral functions with postthrombotic syndrome. Phlebography is no longer universally available.

Recommendations for execution

Please refer to Hach’s recommendations for the execution of ascending phlebography [60, 61].

- Undesired effects/limited interpretability
- Invasiveness of the method
- Radiation exposure
- Possible allergic reactions to the X‑ray contrast medium
- The examination cannot be repeated ad lib
- No conclusions can be drawn for differential diagnosis

An advantage is the ease of documenting the findings without reliance on an observer.

##### Recommendation 27

Phlebography shall not be used in the primary diagnosis of a varicose vein.

#### 5.1.7 Other imaging procedures (endovenous)

Computed tomography (CT)

A common indication for computed tomography (CT) is to clarify veins in the trunk (iliac vein, inferior and superior cava veins, and veins in the pectoral girdle) in the context of a lung embolism diagnosis.

(In-)direct CT phlebography and magnetic resonance phlebography

(In-)direct CT phlebography and magnetic resonance (MR) phlebography are used to view the deep veins and may be indicated in special cases (venous malformations). Computed tomography phlebography should be indicated only after critical consideration because of the radiation exposure involved.

##### Recommendation 28

Computed tomography and MR phlebography shall not be used for the primary diagnosis before treatment of varicose veins.

#### 5.1.8 Other procedures

Phlebodynamometry (PD) is a method for measuring the blood pressure in the peripheral veins and pressure changes in manoeuvre tests and under standardised loads [62]. It is a verifiable method that is highly predictive of venous function. Because it is an invasive procedure (puncture of a vein in the back of the foot), it is not used in everyday routine but is reserved for cases of special doubt.

##### Recommendation 29

Phlebodynamometry should be reserved for special indications.

## 6 Varicose vein treatment

Which treatment strategy is most suitable for each individual case will depend not only on the individual findings but also on the patient’s preference. Patients shall therefore be fully informed about the different options available.

### Recommendation 30

In cases of symptomatic chronic vein disease, the treatment options should always be selected on an individual basis. Invasive procedures, compression treatment, and treatment with drugs are not competing but complementary options; if necessary, a combination of these procedures may be a good course.

### Recommendation 31

The effectiveness of the treatment selected shall be checked regularly against an appropriate parameter (e.g., quality-of-life questionnaire, severity score).

### Recommendation 32

The treating doctor shall distinguish between procedures in which the varicose vein is eliminated and those in which this does not occur.

Possible treatments include the following:

- Conservative measures
  - Compression treatment
  - Physical measures
  - Treatment with drugs
- Operative procedures
  - Saphenous vein ablation procedures
  - Procedures in which the saphenous vein is retained
- Endovenous thermal procedures
  - Endovenous laser treatment
  - Endovenous radio-frequency treatment
  - Endovenous superheated steam treatment
- Endovenous chemical procedures
  - Sclerosis treatment
  - Cyanoacrylate glue

The choice of treatment procedure(s) shall be decided case by case [63]. As a rule, a combination of different measures is recommended; the preferred result is healing of the diseased vein segment [22, 64]. The different methods can be staggered over time or applied in a single session.

### Recommendation 33

The law of minimum invasiveness shall be observed in all measures/interventions. To achieve the object of minimising the invasiveness, a sensible procedure may be a combination of an operation with subsequent sclerosis of tributaries, for example.

### 6.1 Conservative treatment

#### Recommendation 34

Conservative treatment can be considered in all stages of the disease.

It shall be remembered that the effectiveness of conservative measures is limited in certain situations (e.g., in old, multimorbid patients). None of the conservative measures mentioned below can eliminate varicose veins or prevent them from developing; however, they can reduce both the symptoms of the disease and the risk of its evolution and complications.

Conservative treatment includes the following:

- Different types of compression stockings and bandages
- Instrumental intermittent compression/intermittent pneumatic compression (IIC/IPC)
- Other physical decongestion measures, such as
  - Manual lymphatic drainage
  - Balneotherapy
  - Exercise for vascular disease
- Drugs

In addition to putting the leg up and activating the muscle pump in the ankle region by adequate exercise, the basic treatment consists of compression bandages and medical compression stockings; these are designed to improve the venous haemodynamics of the diseased leg.

#### 6.1.1 Compression treatment

**6.1.1.1 Indications.**

##### Recommendation 35

Compression treatment can be applied in all states of varicose veins and chronic venous incompetence. It can be applied alone or in combination with other procedures.

##### Recommendation 36

Clinically significant peripheral arterial occlusion disease and/or advanced peripheral neuropathy (e.g., in cases of diabetes mellitus) impose special demands on the technical execution of compression treatment.

##### Recommendation 37

Selection of the appropriate materials should be guided not only by the indication but also by any comorbidity and the patient’s wishes.

For further details on the different materials, please also see the guideline *“Medical Compression Therapy of the Limbs with Medical Compression Stockings (MCS), Phlebological Compression Bandage Systems (PCB) and Medical Adaptive Compression Systems (MAC).”*

##### 6.1.1.2 Execution.

A summary of the materials

The available treatments are compression bandages, medical compression stockings, and instrumental intermittent compression.

**6.1.1.2.1 Phlebological compression bandage systems (PCB)**

Bandage systems using a single component (e.g., only short-stretch bandages) shall be distinguished from systems using several components (e.g., padding plus short-stretch, short-stretch plus long-stretch). The different materials can be combined individually, or a ready-made system can be used. If different materials are combined, care shall be taken because the general properties of the resulting system, such as the rest and work pressures and the elasticity and stiffness of the material, may alter and will not necessarily be the sum of the individual components. According to the law of Laplace, the pressure under the bandage increases over small radii and can—especially without appropriate inner padding—lead to pressure damage in severe cases [65].

##### Recommendation 38

A compression bandage can be used with inner padding to reduce the risk of severe side effects.

**6.1.1.2.2 Medical compression stockings (MCS)**

A distinction shall be made between medical compression stockings (MCS) and ulcer compression stockings (UCS). Medical compression stockings are classified by compression class (1–4) (RAL 2008), the type of knit (circular or flat), the elasticity of the material (elastic or rigid), and the type (calf stocking, thigh stocking, etc.). They may be mass-produced or made to measure. Because the international classification of compression classes is not universal, when international studies are evaluated, attention should be paid to the absolute pressure rather than to the compression class.

Ulcer compression stockings are also made on the basis of the RAL standard for MCS (RAL 2009); however, they consist of an inner and an outer stocking, which together give a rest pressure of compression class III. The inner stocking can be worn 24 h a day and provides a sliding surface to allow the outer stocking to be pulled on. The outer stocking should be taken off at night. Depending on the supplier, UCS can also be mass-produced or made to measure, and in the latter case fitted with accessories as required.

##### Recommendation 39

For treatment with MCS, the different compression classes and materials should be selected according to the individual needs of the patient.

**6.1.1.2.3 Medical Adaptive Compression Systems (MAC)**

In the last few years, a new type of compression system has become available, which should minimise the donning problems suffered by individual patients with the compression systems available previously [66, 67]. These compression systems are used in the decongestion phase. Like short-stretch bandages, medical adaptive compression systems (MACs) provide a high work pressure and a low rest pressure. In contrast to bandaging systems, loss of pressure can be corrected by adjusting the clips during use, which helps in the remission of oedemas. Because their application is significantly simpler, these systems require less time to apply, and the probability of making a mistake is lower than with more elaborate compression bandaging systems [12]. Patients who are still sufficiently mobile, or whose household members are able, can often apply the MAC themselves after a short introduction. This also improves adherence. Such systems, with their clips, can be taken off and put on, and adjusted as the oedema reduces, relatively easily and independently by sufficiently mobile patients. They are available in different sizes, so MACs can be matched to the patient’s measurements in advance as well as being adjusted at the time of application. There are different systems for treating different indications, such as lymphoedemas, phlebological oedemas, or venous ulcers. Commercially available materials are characterised by relatively high stiffness, which can contribute substantially to their effectiveness. A MAC can be used alone or in combination with MCS. Patients can handle these systems themselves, as they have reproducible pressures and generally a high level of stiffness.

##### Recommendation 40

In the initial decongestion phase with lymphoedema and pronounced venous oedema, as well as venous leg ulcer, a MAC can be applied as an alternative to bandaging.

**6.1.1.2.4 Intermittent pneumatic compression (IPC)**

Intermittent pneumatic compression (IPC) systems consist of a compressor/control unit and leg or trouser cuffs for the varicose vein. The compressor is programmed with the treatment time, desired work pressure, and the inflation, plateau, and deflation times, depending on the type of equipment. An IPC system is a listed aid and can be used by both bedridden and ambulatory patients in the clinic and/or at home. A test phase is recommended before equipment is ordered for home use.

For further details about IPC, please see the corresponding guidelines (intermittent pneumatic compression [IPC, IIC]).

To date there are no mandatory treatment protocols for cycle design, treatment time, treatment pressure, or treatment frequency. The data available on the use of IPC for venous oedemas and ulcers are inconsistent. The following recommendation is based on the available literature and the consensus of experts.

##### Recommendation 41

Intermittent pneumatic compression can be used in patients with venous oedemas and/or dermatoliposclerosis to reduce painful venous symptoms.

##### 6.1.1.3 Use.

Compression treatment can improve venous symptoms and alterations such as venous oedema [68]. The strongest evidence and the broadest basis for compression treatment exists for advanced CVI, especially CEAP states C5 and C6 [69].

**6.1.1.3.1 Venous symptoms**

Compression treatment reduces venous symptoms such as the feeling of heaviness, paraesthesia, and tendency to swelling (especially after sitting or standing for a long period), even with a low rest pressure of < 20 mm Hg [68, 70–73].

##### Recommendation 42

All patients with venous symptoms (CEAP classes C1s–C6) shall receive compression treatment to relieve the symptoms.

**6.1.1.3.2 Venous oedema**

Compression treatment reduces venous oedema in patients with a varicose vein/CVI [74]. The higher the resting pressure and the firmer the material, the more effective the treatment is in reducing the oedema. However, even so-called placebo stockings with a pressure of < 10 mm Hg have been shown to be able to reduce or prevent oedema. Compression treatment with low (10–20 mm Hg) or medium pressure (20–30 mm Hg) can be applied as a prophylactic against venous oedema [75]. Patients with very pronounced oedemas have used compression bandages for initial decongestion, either with different single components or in combination.

##### Recommendation 43

Compression treatment should be applied to the leg with the oedema in two phases (decongestion phase and maintenance treatment).

##### Recommendation 44

Slight oedemas can be treated by compression directly with medical compression stockings.

**6.1.1.3.3 Skin alterations**

Compression treatment also reduces lipodermatosclerotic skin alterations in patients with varicose veins/CVI [76].

**6.1.1.3.4 Venous leg ulcer**

There is abundant evidence for compression treatment in the treatment and prophylaxis of venous leg ulcers. Compression treatment speeds up the healing of a venous leg ulcer (UCV) or a mixed leg ulcer [69, 77–79]. It also extends the period before recurrence and decreases the frequency of recurrence [69, 78]. Compression treatment with high pressure (at least MCS class 2) appears to be more effective both for treatment and as prophylaxis against recurrence than compression treatment with a lower pressure [78, 80]. Nevertheless, even a low rest pressure (equivalent to compression class [CCL] 1, 18–21 mm Hg) leads to healing and reduced recurrence of UCV [77]. There is, however, a lack of studies comparing no compression with compression treatment as a prophylactic against recurrence [81].

The effectiveness and value of a compression bandage depend on the ability and experience of the person applying it every time it is applied [75]. A well and expertly applied compression bandage is still a very effective measure, even today [75]. In everyday practice, however, we often come across very poorly applied compression bandages, which very quickly lose their initial rest pressure, slip, and produce side effects.

In comparative studies, UCS have proved better than compression bandages with short-stretch bandages for pain reduction, ulcer healing, and frequency of recurrence [82, 83]. For multiple component bandages, however, no significant differences were found between bandages and UCS [84]. A UCS system with a thigh-length inner stocking presents advantages over a system with a lower leg stocking in terms of volume reduction and venous haemodynamics [85].

In a literature review, the healing rates of venous leg ulcers with properly applied compression bandages were comparable to those with UCS. Everyday handling of UCS (other than in studies) is often easier, and incorrect application is less likely to occur. The use of IPC in addition to static compression treatment has proved helpful in venous leg ulcers (see IPC guidelines).

##### Recommendation 45

Compression bandages shall be applied by properly trained persons.

**6.1.1.3.5 Prophylaxis against CVI**

Chronic venous insufficiency (CVI) is known to be a chronic-progressive disease, the causes of which have not yet been fully explained. On the basis of current knowledge, therefore, the optimum goal of treatment is not to cure the disease but to reduce its painful symptoms and prevent complications.

As noted previously, there are solid data in the literature that show that compression treatment produces good results in relieving symptoms, curing venous ulcers, and preventing their recurrence. However, there are insufficient data to prove that the use of compression treatment prevents the progression of CVI.

**6.1.1.3.6 Postinterventional compression treatment**

**6.1.1.3.6.1 Indications**

Postinterventional compression treatment comprises treatment after stripping operations, tributary removal, endovenous thermal procedures, and both foam and liquid sclerotherapy. The object of postinterventional compression treatment is to reduce treatment-associated symptoms such as pain, oedema, haematoma, and numbness and to improve the clinical outcome.

**6.1.1.3.6.2 Liquid sclerotherapy**

Randomised controlled studies have shown that the use of MCS over a period of 3 weeks significantly improves the effectiveness of liquid sclerotherapy of spider veins and reticular varices [86–88]. There are indications that compression can reduce the frequency of side effects, especially hyperpigmentation [88].

##### Recommendation 46

Compression treatment should be applied after liquid sclerotherapy of spider veins and reticular varices, as it improves the clinical response and reduces hyperpigmentation. The duration of compression treatment can be established individually.

**6.1.1.3.6.2** **Foam sclerotherapy**

The existing recommendations to apply compression treatment after foam sclerotherapy have grown up on a largely historical and regionally specific basis [89]. Painful inflammatory reactions and superficial vein thrombi often occur in tributaries after sclerotherapy. So far there is no sufficient evidence of the effectiveness of compression treatment against complications, which affect tributaries in particular. Whether compression treatment improves the response to the treatment has not yet been investigated.

##### Recommendation 47

Compression treatment shall be applied after foam sclerotherapy of varicose saphenous veins and tributaries. The type and duration of the treatment can be established individually.

**6.1.1.3.6.3 Open operative treatment of the varicose vein**

The outcome of a meta-analysis of the duration of compression treatment after surgical vein treatment was that there was no difference in postoperative pain, leg volumes, frequency of complications, or length of sick leave between the short-term compression group (3–10 days) and the long-term group (3–6 weeks) [90, 91].

On the assumption that higher local pressures have a positive effect on postoperative pain, Benigni et al. investigated the effect of a compression pad under a compression stocking after GSV stripping. Postoperative pain was significantly reduced in the group with a compression pad [92]. Similar results were also reported by Lugli et al. after endovenous laser treatment [93].

Reich-Schupke et al. showed that after crossectomy and stripping, compression stockings with a pressure of 23–32 mm Hg were significantly superior to stockings of 18–21 mm Hg in the regression of oedemas, pain, and feelings of tension and discomfort in the leg in the first few weeks [94]. With respect to the choice of compression material, Mariani et al. showed in an investigation that patients who were supplied with compression stockings after an operation presented significant regression of oedemas and better acceptance and quality of life compared with patients who received compression bandages [95].

The current recommendations of the Society for Vascular Surgery and the American Venous Forum support postoperative compression treatment after a stripping operation. This should be continued for at least 1 week [96].

**6.1.1.3.6.4 Endovenous thermal procedures**

A systematic review of compression treatment after endovenous ablation showed that the strategies and recommendations for postoperative compression treatment after endovenous ablation are very variable and not based on evidence but rather on the authors’ experience [97]. A randomised controlled study published by Ayo et al. showed that postoperative compression treatment with 30–40 mm Hg for 7 days did not produce differences either in the clinical outcome or in patient reports compared with a control group without any compression [98].

Bakker et al., on the other hand, came to the opposite conclusion. The authors investigated to what extent postoperative compression with 32 mm Hg after isolated laser ablation of the GSV, applied for more than 48 h, produced an advantage in quality of life and postoperative morbidity. They showed that after 1 week, the group who wore compression for the whole week presented significantly less pain and significantly better functionality and vitality than the group who wore compression only for 48 h. This difference was no longer detected after 6 weeks [99].

Lugli et al. showed that eccentric compression after endovenous laser ablation significantly reduced postoperative pain in the first week of treatment (*p* < 0.001) [93].

The current recommendations of the Society for Vascular Surgery and the American Venous Forum support postoperative compression treatment for at least 1 week after endovenous laser ablation [96].

##### Recommendation 48

After an operation or endovenous thermal treatment of the superficial vein system, initial postoperative/postinterventional compression treatment should be applied. The type and duration of the treatment can be established individually. Eccentric compression in the thigh can be applied for pain reduction after GSV interventions.

**6.1.1.3.6.5 Long-term compression after invasive varicose vein treatment**

There are no randomised controlled studies that show improvement in the response to treatment as a result of long-term compression treatment.

The greatest lack is in study design and in the length of the posttreatment observation period. Individual studies have not yet confirmed the advantage of long-term compression [89, 93, 100–102].

If symptomatic venous disease persists despite invasive treatment, further compression treatment is indicated [103]. Compression should also be continued if a venous functional deficit persists [104].

##### Recommendation 49

Patients in whom residual symptoms of chronic venous incompetence persist despite invasive treatment should receive continued compression treatment.

Long-term compression treatment is not recommended to improve the clinical response after invasive vein treatment.

**6.1.1.3.7 Selection of material**

It is known that the effect of compression even at low pressures (class I, 18–21 mm Hg) helps to reduce or eliminate oedemas [72, 105], improves venous ejection [106], allows venous ulcers to heal [69], and reduces the recurrence rate of new ulcers [78].

Patient adherence falls as the resting pressure of the compression treatment increases, regardless of the indication that led to the compression treatment [81, 94]. To date there is little evidence for special compression pressures to meet different indications. Whenever possible, therefore, the lowest medically justifiable resting pressure should be selected.

The working pressure appears to be the most important parameter for the venous ejection fraction and thus for the effectiveness of compression treatment. This may be influenced on the one hand by the resting pressure and on the other by the firmness and elasticity of the material [77, 106]. It has been shown that the working pressure of the compression material can be achieved not only by increasing the resting pressure but also by reducing the elasticity of the material.

##### Recommendation 50

To improve patient adherence, the compression treatment shall be applied using the lowest medically justifiable resting pressure. The prescribed resting pressure is established on an individual basis.

##### 6.1.1.4 Aids.

Handling their own compression equipment may cause patients difficulties; they may even find it impossible, depending on their comorbidities [107]. To ensure correct treatment, these patients will need people to help them (e.g., relatives, nursing staff) or will need aids [107, 108].

**6.1.1.4.1 Aids for MCS**

Aids for putting on and taking off **medical compression stockings** include rubber gloves, slip-socks, frames, and unrolling aids. These make handling demonstrably easier, especially for elderly and obese patients [108]. Aids for putting on and taking off **medical compression stockings** are ordered as a special prescription. It is helpful to provide the justification and the indication.

##### Recommendation 51

Patients should be offered aids when they are prescribed MCS.

##### Recommendation 52

If the patient cannot put on or take off the MCS by herself/himself even with these aids, the help of another person(s) should be incorporated. The person(s) should be trained in compression treatment practices.

**6.1.1.4.2 Aids for bandages**

There are no aids to allow patients to put on their own compression bandages. In principle, it is possible to teach patients to put on their own bandages; however, applying a properly wrapped compression bandage to one’s own leg is very difficult, even for a trained individual.

##### 6.1.1.5 Improving adherence.

Patient adherence to compression treatment is often less than optimal. In cases of a severe indication, e.g., venous leg ulcer or postthrombotic syndrome, consistent and complete application of the treatment is essential. There are different measures available to help patients adhere to their treatment. These include programmes such as leg ulcer clubs, nurses to teach self-management, and educational materials for patients in video or text form. However, there is no clear, up-to-date assessment of the effects of these measures on the healing and recurrence rates of venous leg ulcers. Further studies are necessary to evaluate the effects of these measures [109]. Full explanations and repeated recommendations of compression treatment by doctors appear to have a positive impact on adherence to compression treatment by patients with CVI [90].

##### 6.1.1.6 Side effects.

Correctly indicated and executed compression treatment is a safe and effective measure. Dryness and scaling of the skin can be expected on a regular basis as a consequence of the drying effect of compression treatment; these conditions can be relieved with suitably frequent skin care, which should form a part of any compression treatment.

##### Recommendation 53

All patients with compression treatment shall be recommended to apply skin care, however long the treatment period and whatever the treatment indication.

Allergic reactions to compression stockings are rare [110]. If clinical suspicion arises of a contact allergy in CVI patients, a patch test applied by an allergy specialist is recommended. In allergic contact eczema, recognition of the allergen is decisive, as the allergy can be cured only after the allergen is removed. Apart from the standardised test series, it is useful to consider any textile tests brought in by patients. The most significant factors, apart from isolated textile allergies, are natural latex, textile additives (e.g., formaldehyde), and dyes [111].

Other possibilities, mostly involving mishandling or poor skin care, are nerve damage, circulation disorders, erosion and ulcers, itching, reddening of the skin, eczema, paraesthesia, feeling hot or cold, sweating, constriction, and thrombosis.

#### 6.1.2 Treatment with drugs

##### 6.1.2.1 Indication.

**Fig. 1:**
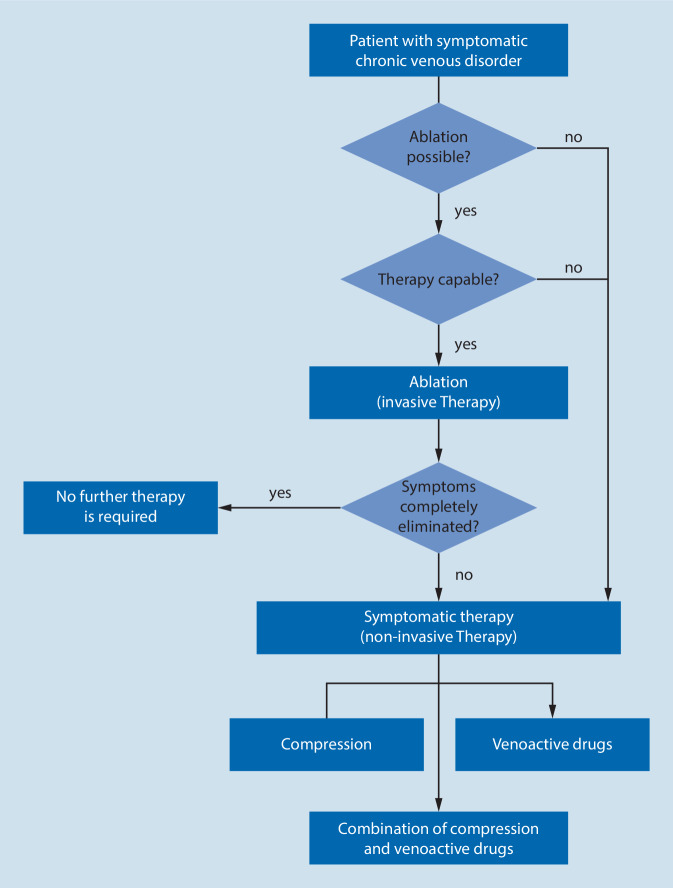
The treatment or combination of treatments for symptomatic chronic vein disease shall be selected on an individual basis, determined by different factors (from [103])

If it is impossible or considered undesirable to treat a symptomatic varicose vein, or if swelling and feelings of heaviness persist even after invasive treatment, the prescription of oral vein treatment drugs of proven effectiveness can be considered ([96, 103]; Fig. 1).

##### Recommendation 54

If it is impossible or considered undesirable to treat symptomatic varicose veins, or if a symptomatic venous picture persists even after invasive treatment, conservative treatment with effective venous drugs can be applied.

##### Recommendation 55

In cases of chronic disease and persistent symptoms, long-term conservative treatment should be applied.

The different conservative principles complement one another: While the venous reflux is controlled by the external pressure of the compression elements, plant-based vein drugs work on the vessel walls and reduce their permeability. Combining the two procedures appears to strengthen these effects by synergy [112, 113].

##### Recommendation 56

If single treatment procedures do not produce the desired relief, a combination of the different treatment principles (drug treatment, varicose operation, compression treatment) can be considered.

##### 6.1.2.2 Guidelines for application.

In Germany, the following drugs with evidence-based effectiveness are available for oral treatment: standardised red vine leaf extract (AS 195), standardised horse chestnut extract, and oxerutin. Other drugs of proven effectiveness (e.g., certain ruscus-containing vein tonics, troxerutin–coumarin combinations) are currently not approved in Germany (drug information system [AMIS] of the German drug authorisation agency *Arzneimittelzulassungsbehörde* 2015). Plant-based vein drugs reach their maximum effectiveness only after a certain period of continuous administration of 2–4 weeks. This should be explained to the patient [10, 74, 112, 114–125].

##### Recommendation 57

It should be explained to the patient that the clinical impact of the full effectiveness of plant-based vein drugs can generally be assessed only after a certain period of continuous administration of 2–4 weeks.

##### 6.1.2.3 Results.

Controlled studies are available for the substances approved for use in Germany, namely standardised red vine leaf extract (AS 195), standardised horse chestnut extract, and oxerutin. They document both the regression of oedemas and relief of subjective painful symptoms: pain, feelings of heaviness, and tension. The clinical effects are based on the fact that the active ingredients of the drug have an anti-inflammatory effect and normalise the permeability of venous vessels [10].

The symptomatic effectiveness is documented with good evidence for different products [10, 74, 112, 114–118, 120–126]. Standardised red vine leaf extract (AS 195), standardised horse chestnut extract, and oxerutin significantly reduce oedemas. Furthermore, red vine leaf extract and oxerutin have also been shown to produce significant improvement in symptoms [115–117, 120–122, 127, 128].

Table 3 summarises the evidence for the vein drugs approved in Germany. Apart from randomised studies, which formed the source for the above criteria, supporting data have also been provided by small or short studies.

**Table 3:** Summary of data for the vein drugs approved in Germany (from [103])

Active principle	Dose	Data from robust RCTs^a^		Supporting data^b^
		Significant reduction of oedema	Significant improvement in symptoms	
Standardised red vine leaf extract^c^	Once per day 360–720 mg	Kiesewetter et al. (2000) [120]	Kiesewetter et al. (2000) [120]	Kalus et al. (2004) [123]
		Rabe et al. (2011) [121]	Rabe et al. (2011) [121]	
Standardised horse chestnut extract^d^	Twice per day 50 mg (aescin)	Diehm et al. (1996) [74]	n. i.	Neiss et al. (1976) [124]
				Cloarec (1992) [117]
Oxerutin	Twice per day 500 mg	Unkauf et al. (1996)^e^ [112]	n. s.^e^	Cloarec et al. (1996) [125]
		Diebschlag et al. (1994) [122]	Diebschlag et al. (1994) [122]	Grossmann et al. (1997) [118]
				Petruzzellis et al. (2002) [114]

*n.* *i.* not investigated, *n.* *s.* not significant, *RCT* randomised controlled trial

^a^Carried out in accordance with the guidelines of the German Society of Phlebology (DGP). Vanscheidt et al. (2000) [119]

^b^Small and/or short RCTs, inadequate measurement of the reduction of oedemas, etc.

^c^Proportion of active principle red vine leaf extract (RVLE); 4–6:1

^d^Proportion of active principle horse chestnut extract (HCSE); 4.5–5.5:1

^e^Compression therapy in both treatment groups

The study findings for plant-based drugs shall always be strictly limited to the drugs actually tested. This is true of both red vine leaf extracts and horse chestnut extracts. Standardised production procedures shall be demanded for plant extracts [103].

**6.1.2.4 Side effects.**

##### Recommendation 58

Drugs shall not be used in patients who have a known hypersensitivity (allergy) to the drug in question.

##### Recommendation 59

Attention shall be paid to the following side effects: The principal side effects described for oral administration of the drugs mentioned above are intestinal irritation and skin reactions. If these effects occur, a change to one of the other two evidence-based vein drugs should be considered.

#### 6.1.3 Physiotherapy

##### 6.1.3.1 Manual lymphatic drainage.

Some of the lymph collectors in the leg lie close to the saphenous veins [129]. Lymphatic incompetence is not infrequently induced by venous incompetence; it may improve after treatment of the varicose vein [130]. There are case reports stating that manual lymphatic drainage can reduce treatment-resistant oedemas associated with a varicose vein.

##### Recommendation 60

Manual lymphatic drainage can be considered in cases of venous oedema associated with a varicose vein, if other treatment options such as compression treatment, invasive treatment of the varicose veins, and treatment with drugs do not sufficiently reduce the venous oedema.

### 6.2 Invasive varicose vein treatment

Incompetent saphenous veins and accessory veins, varicose tributaries, and incompetent perforator veins can be treated either by open surgery or endovenously.

In most cases, a saphenous varicose vein does not occur in isolation but in combination with a varicose tributary. After ablation of the varicose saphenous vein, the diseased tributary may persist or disappear.

#### Recommendation 61

In treatment planning, it shall be considered that the incompetence of the tributary may resolve itself after removal of the saphenous vein.

#### Recommendation 62

Removal of a diseased tributary can be carried out simultaneously with treatment of the saphenous vein, or subsequently.

Anaesthesia is required with most methods. The spectrum ranges from general anaesthesia through peridural anaesthesia to local anaesthesia. In many cases, tumescence, and particularly tumescent local anaesthesia, plays a significant role or is even an integral constituent of the method. Some particularities shall be observed.

*Tumescent local anaesthesia in varicose vein treatment*

In endovenous thermal varicose vein ablation, the strongest recommendation is for the introduction of a perivasal liquid depot to allow vein ablation with minimal collateral damage and avoidance of burns.

Tumescent local anaesthesia (TLA) can be used concurrently to ensure sufficient analgesia. It involves the subcutaneous infiltration of a very diluted local anaesthetic in a buffered solution with added adrenalin [131, 132]. Omitting any individual component should be avoided due to alterations to the pharmacokinetics [133].

#### Recommendation 63

In invasive procedures that require the use of a tumescent solution, the combined application of a local anaesthetic can avoid a second anaesthetic procedure.

#### Recommendation 64

In operations, TLA can be used alone or together with other anaesthetic procedures.

#### Recommendation 65

Omitting any individual component of the TLA solution should be avoided because of alterations to the pharmacokinetics.

In addition to postoperative maintenance analgesia, TLA can be advantageous for intraoperative mobilisation (hydrodissection) of the vein to be ablated and for reducing the formation of postoperative haematomas [134].

When TLA is used, the recommended limit doses of the local anaesthetics used—although they are not strict limits—are knowingly exceeded [135, 136].

Discussion about the off-label use of TLA has developed in the absence of clear authorisation of the maximum joint dosage in TLA [137]. It should be noted that preparation of the TLA solution requires the production of at least an infusion solution and possibly also a drug [138]. The pertinent hygiene provisions shall be observed. Even with small amounts, the status of off-label use is subject to legal discussion.

#### Recommendation 66

The use of TLA involves off-label use. This shall be explained to the patient.

#### 6.2.1 Open operative procedure

##### 6.2.1.1 Bases.

Different strategies are available for open operative treatment of varicose veins. In addition to vein removal procedures (e.g., crossectomy, stripping, phlebectomy), vein-conserving concepts also exist (e.g., the CHIVA treatment, extraluminal valvuloplasty). In the presence of trophic disorders, special surgical procedures (e.g., shave treatment, fascia surgery) may be necessary (see the guidelines for the treatment of venous leg ulcer) [139–144].

##### Recommendation 67

Depending on the individual indication, the following open operative procedures shall be applied: vein removal methods (e.g., crossectomy, stripping, phlebectomy) and vein-conserving concepts (e.g., CHIVA, extraluminal valvuloplasty).

##### Recommendation 68

In the presence of trophic disorders, special surgical procedures (e.g., shave treatment, fascia surgery) can be necessary.

**6.2.1.1.1 Indications**

In principle, all forms of varicose vein (except spider veins) characterised by reflux detectable by duplex ultrasound can be treated with an open operational procedure.

The reflux source shall be identified unequivocally before the operation. It may be in the region of the saphenofemoral/saphenopopliteal junction (SFJ/SPJ), a perforator vein, or the pelvic region. The treatment concept shall focus on the reflux source.

A special indication for the operation can result from complications of the varicose vein [145, 146]. These include superficial vein thrombosis, including ascending varicophlebitis [19, 147–151] and variceal bleeding [152].

In cases of a secondary varicose vein or angiodysplastic alterations, the ablation of incompetent superficial veins may be needed to improve venous haemodynamics. Definition of the indication assumes proper diagnosis, excluding a collateral function of the vein to be treated [153, 154].

##### Recommendation 69

The following indications for varicose vein operations shall be followed: saphenous varicose veins, accessory varicose veins or tributaries, recurrent varicose veins, varicose veins with venous angiodysplasia, superficial vein thrombosis, variceal bleeding.

##### Recommendation 70

The treatment concept of the varicose vein operation shall focus on the proximal reflux source.

**6.2.1.1.2 Contraindications for elective operations on superficial varicose veins**

The following absolute and relative contraindications apply to varicose vein operations [155–158].

Absolute contraindications:

- Acute thrombosis of the deep leg vein and/or iliac vein
- Peripheral arterial occlusion disease from Fontaine stage III (except by special indication)
- Known pregnancy
- Moribund patient (ASA score 5)

##### Recommendation 71

The following absolute contraindications shall be observed in open varicose vein operations: acute thrombosis of the deep leg vein/iliac vein, peripheral arterial occlusion disease from Fontaine stage III (except by special indication), known pregnancy, and moribund patient (from American Society of Anesthesiology [ASA] score 5).

Relative contraindications:

- Peripheral arterial occlusion disease from Fontaine stage IIb
- Serious disturbance of haemostasis
- Severe lymphoedema
- Very severe general disease (from ASA score 4)

##### 6.2.1.2 Operations to remove varicose veins.

The principle of the classic operative treatments of varicose saphenous veins consists of interrupting the reflux at the proximal and distal reflux sources, selectively removing incompetent sections of the superficial vein system (interrupting the recirculation circuit according to Hach), and thus achieving the longest-lasting normalisation possible of the venous haemodynamic [159–163]. The literature shows that operative removal shall be dependent on the existing state [160, 163–167].

The varicose vein operation can consist of several components in combination, depending on the expression in the findings [151, 157, 159, 168–172].

##### Recommendation 72

Operative removal should be limited to the diseased vein segments (depending on the existing state).

**6.2.1.2.1 Recommendations for execution of surgical procedures to remove varicose veins**

The success of operative measures in the superficial vein system is significantly conditioned by preoperative planning with duplex ultrasound support to mark the incompetent veins (mapping). Mapping helps to reveal any anatomical variations and to define the treatment strategy; it should preferably be carried out personally by the operating surgeon.

##### Recommendation 73

Before vein surgery, the incompetent vein segments should be identified in duplex ultrasound and marked on the skin (mapping).

**6.2.1.2.2 Interrupting the incompetent transfascial communication(s)**

1. Flush ligation of the saphenofemoral junction (crossectomy) of the GSV
2. Flush ligation (or as near to the saphenopopliteal junction as possible) of the small saphenous vein (SSV)
3. Ablation of incompetent perforator veins

**6.2.1.2.2.1 Crossectomy of the great saphenous vein (GSV)**

Crossectomy is the flush ligation of the great saphenous vein at the saphenofemoral junction (SFJ), interrupting all the tributary veins that drain into the GSV in the junction region with resection of the GSV itself near the SFJ [159, 165, 173–195].

The use of absorbable or nonabsorbable suture is controversial and is discussed in the literature [173, 196]. At all events, the use of absorbable materials for ligation seems to be associated with more frequent appearance of veins carrying reflux at the ablation point in the junction region [197]. In the past, the use of nonabsorbable suture was normal in up to two-thirds of interventions, as a survey among varicose vein surgeons in Germany, Austria, and Switzerland 20 years ago showed [198]. No disadvantages are known to result from this practice. Nonabsorbable ligation material can therefore be recommended for the SFJ region as the simplest (and cheapest) solution to prevent recurrence [199, 200].

The tributaries that flow into the deep vein in the SFJ region should be interrupted separately [175, 178, 179, 201, 202].

##### Recommendation 74

To prevent recurrence in the groin region, the crossectomy shall be free of technical imperfections; the use of nonabsorbable suture can be recommended.

**6.2.1.2.2.2 Ligation of the small saphenous vein (SSV) at the saphenopopliteal junction (SPJ)**

The SPJ region is extremely variable [203–207] and may be difficult to display; flush ligation is very often possible, but not always [155, 175]. To reduce the frequency of saphenopopliteal recurrence, the vein should be interrupted as close as possible to the junction [208–212].

The question as to whether muscle veins that flow into the SPJ should also be interrupted is not yet clear. To prevent hernias, every effort should be made to close the fascia.

##### Recommendation 75

To reduce the frequency of saphenopopliteal recurrence, the vein should be interrupted as close as possible to the saphenopopliteal junction.

**6.2.1.2.2.3 Ablation of perforator veins**

In the context of surgical ablation of varicose tributary veins, in individual cases it may be necessary also to interrupt a perforator vein [213–217]. The ablation of incompetent transfascial connecting veins can help prevent or cure trophic skin damage [172, 218–220].

##### Recommendation 76

Perforator veins should be eliminated when they form the proximal reflux source.

##### Recommendation 77

In cases of clinical/haemodynamic importance with an incompetent or obstructed deep vein system, the interruption of incompetent perforator veins can be considered.

The following operation techniques are available:

- Direct epifascial or subfascial interruption
- Subfascial endoscopic perforating vein surgery (SEPS) [141, 221–226].

Subfascial endoscopic perforating vein surgery (SEPS) should be indicated only very reluctantly, and never in cases of ulcer surgery because of the high morbidity rate [221, 227].

##### Recommendation 78

When indicated, the perforator veins should be interrupted by epifascial or subfascial intervention.

##### Recommendation 79

Subfascial endoscopic perforating vein surgery shall be used only in individual cases and not carried out routinely.

**6.2.1.2.2.4 Ablation of diseased segments of saphenous vein**

The saphenous veins (GSV and/or SSV) can be eliminated either completely or partially, depending on where the proximal and distal reflux sources are [4, 157, 228–233]. In incomplete forms of varicose GSV, where the proximal reflux source lies distal to the SFJ region, partial elimination is generally sufficient [234]. Healthy vein segments should be retained [155, 156, 165, 235, 236].

Elimination of a saphenous vein down to the ankle region is associated with a high incidence of sensory nerve disturbances [167, 237–239].

There are various operative methods for removing a diseased segment of saphenous vein (e.g., conventional stripping, invaginated stripping, cryostripping, extraluminal stripping, phlebectomy). The direction of stripping can be from distal to proximal or from proximal to distal. Sensory nerve lesions are less likely with the frequently used proximal-to-distal invaginated stripping method [240]. The literature does not indicate that any particular method is superior to the others [241–248].

##### Recommendation 80

Various methods can be used for stripping the diseased vein segment, e.g., conventional stripping, invaginated stripping, cryostripping, extraluminal stripping, phlebectomy.

##### Recommendation 81

Carrying out the stripping manoeuvre from proximal to distal may be preferable, as fewer sensory nerve lesions are observed.

##### Recommendation 82

During postoperative care, the outcome of the operation (removal of the diseased saphenous vein) should be confirmed by duplex ultrasound.

**6.2.1.2.2.5 Ablation of diseased tributaries**

Tributary ablation is carried out through very small incisions in the skin with fine hooks or specially made instruments [157, 249, 250].

##### Recommendation 83

In cases of isolated tributary reflux, phlebectomy can be used as the only method.

##### Recommendation 84

Combining phlebectomy with simultaneous or subsequent sclerotherapy can be a satisfactory treatment.

**6.2.1.2.3 Undesired effects and complications**

If correctly carried out, open operative treatment of varicose veins is a safe form of treatment with few side effects. The following undesired effects and complications are rare; however, they may sometimes occur and be observed [155, 163, 251–260].

**6.2.1.2.3.1 Intraoperative complications:**

- Bleeding: 0.01%–0.1% [257, 261, 262]
- Nerve damage: 0.01%–6.6% [172, 229, 240, 257, 263–269]
- Injury to the lymph vessels: individual cases [253, 270, 271]
- Injury to large vessels: 0.01%–0.1% [156, 172, 253, 257, 272–275]

##### Recommendation 85

Special attention shall be paid to the following possible intraoperative complications in open operative treatment of varicose veins: bleeding, injury to large vessels, nerve damage.

**6.2.1.2.3.2 Postoperative complications:**

- Secondary bleeding, haematoma: 0.06%–2.0% [159, 258, 265, 276, 277]
- Wound infection: 0.1%–2.8% [272, 277, 278]
- Wound healing disturbances: 0.05%–1.38% [258, 272]
- Pigmentation disturbances: individual cases [279]
- Superficial vein thrombosis: 0.2%–0.3%
- Lymphatic fistula, lymphatic cyst, lymphoedema: 0.02%–1.82% [159, 257, 258, 280]
- Necrosis: individual cases [258, 278]
- Deep leg vein thrombosis and/or lung embolism: 0.01%–0.24% [251, 257, 258, 265, 272, 277, 281]
- Occurrence of spider veins, matting: individual cases
- Pathological scar formation: individual cases
- Compartment syndrome: individual cases

##### Recommendation 86

Special attention shall be paid to the following possible postoperative complications in open operative treatment of varicose veins: secondary bleeding/haematoma, wound infection/healing disturbances, lymph vessel disturbances, superficial/deep leg vein thrombosis, lung embolism.

Death has been reported in isolated cases: 0.004%–0.023% [258, 277, 282].

The frequencies of the individual complications in over 150,000 interventions are shown in Table 4.

**Table 4:** Combined total of complications/side effects of open operative treatment of varicose veins (absolute/%): 151,720 legs operated, nine studies, 1983–2013

**Author-**	**All**	Helmig		Balzer		Hagmüller		Nüllen		Frings		Critchley		Hofer		Noppeney		Papapostolou	
**Year**	–	1983 [258]		1983 [282]		1992 [257]		1995 [283]		1995 [284]		1997 [265]		2001 [259]		2005 [153]		2013 [252]	
**Number of “cases”**	–	–		25,457		–		–		47,057		–		–		–		–	
**Patients**	–	13,024		–		–		1683		–		599		1094		36,323		841	
**Legs**	–	20,353		–		3300		1981		–		973		1590		49,939		1070	
–	**%**	***n***	**%**	***n***	**%**	***n***	**%**	***n***	**%**	**nn**	**%**	**nn**	**%**	**nn**	**%**	**nn**	**%**	**nn**	**%**
**Intraoperative/intraprocedural**																			
*Bleeding/Shock*	**0.01–0.1**	1	0.005	4	0.015	–	–	13	0.66	–	–	–	–	–	–	–	–	–	–
*Transfusion*	**0.05**	–	–	–	–	–	–	–	–	–	–	–	–	–	–	23	0.046	–	–
*Injury to large arteries*	**0.01**	1^a^	0.005	3	0.012	–	–	–	–	1^b^	0.002	1^b^	0.10	–	–	1	0.002	–	–
*Injury to large veins*	**0.01–0.1**	10	0.049	2	0.008	1	0.03	–	–	4	0.009	–	–	–	–	21	0.042	–	–
*Nerve damage* – Motor	**0.01–0.1**	2^c^	0.010	–	–	1	0.03	–	–	3^c^	0.006	1^c^	0.10	–	–	6	0.012	–	–
– Sensory	**0.2–6.6**	43	0.21	–	–	–	–	95	4.8	–	–	64	6.58	18	1.13	48	0.096	34	3.18
**Postoperative/postprocedural**																			
*Secondary bleeding*	**0.06–0.1**	–	–	15	0.059	–	–	2	0.1	–	–	–	–	–	–	–	–	–	–
Requiring revision	**0.13**	26	0.13	–	–	–	–	–	–	–	–	–	–	–	–	–	–	–	–
Requiring transfusion	**0.06–0.21**	42	0.21	–	–	–	–	–	–	–	–	–	–	–	–	32	0.06	–	–
*Haematoma*	**0.6–2.0**	130	0.64	–	–	–	–	35	1.77	–	–	–	–	30	1.89	585	1.17	6	0.56
Puncture/surgical haematoma removal	**0.1**	25	0.12	–	–	–	–	–	–	–	–	–	–	–	–	–	–	1	0.093
*Suffusion/ecchymosis*	–	–	–	–	–	–	–	–	–	–	–	–	–	–	–	–	–	–	–
*Seroma*	**1.4**	–	–	–	–	–	–	–	–	–	–	–	–	–	–	–	–	15	1.4
*Wound infection*	**0.1–2.8**	25	0.12	13	0.051	–	–	1	0.05	72	0.153	27	2.77	21	1.32	387	0.78	23	2.15
With revision	**0.22**	–	–	–	–	–	–	–	–	–	–	2	0.21	–	–	115	0.23	–	–
*Wound healing disturbance*	**0.05–1.38**	232	1.14	–	–	–	–	1	0.05	–	–	–	–	22	1.38	–	–	–	–
*Pigmentation disturbance*	**0.2**	–	–	–	–	–	–	–	–	–	–	–	–	–	–	–	–	2	0.18
*Lymphatic fistula/cyst/oedema*	**0.02–1.82**	112	0.55	16	0.063	–	–	3	0.15	–	–	8	0.82	29	1.82	–	–	–	–
*Superficial vein thrombosis*	**0.2–0.3**	–	–	–	–	–	–	–	–	–	0.049	2	0.21	–	–	146	0.29	2	0.18
*Deep leg vein thrombosis*	**0.03–0.24**	11	0.05	7	0.027	8	0.24	2	0.1	23	–	3	0.31	2	0.13	39	0.08	1	0.093
*Lung embolism*	**0.01–0.13**	2	0.01	5	0.020	2	0.06	–	–	6	0.013	1	0.10	2	0.13	8	0.02	–	–
**Other complications**^**d**^	**0.1–4.2**	22	0.11	–	–	–	–	83	4.19	–	–	4	0.41	–	–	56	0.11	1	0.093
**Deaths**^**e**^	**0.004–0.023**	2	0.005	1	0.004	–	–	–	–	–	–	–	–	–	–	–	–	–	–

Injury to large arteries/veins:

^a^Very thin female patient

^b^During revision of saphenofemoral junction (SFJ) treatment

^c^During saphenopopliteal junction (SPJ) operation, some with course of the nerve in the operation region described as complicated

^d^Other complications: Helmig: increase of a preexisting swelling (during diagnosis of possible deep vein thrombosis [DVT]); Nüllen: severe oedema; Papapostolou: suture granuloma; Critchley: blisters due to compression bandage, breast infection (?); Noppeney: other intraoperative complications

^e^Deaths: Balzer: lung embolism; Helmig: 1 × ventricular fibrillation under general anaesthesia, 1 × warfarin bleeding after 9 months (due to thrombus)

**6.2.1.2.4 Outcomes**

All the currently available studies show a clear improvement in postoperative quality of life after open varicose vein surgery, in both general and disease-specific terms. This effect persists for more than 5 years [185]. The recurrence rate over time is an important parameter for the outcome in terms of postoperative quality of life [285].

Outcome studies shall distinguish between technical (duplex, reflux finding) and clinical recurrence (visible varicose veins, painful symptoms, quality of life) [185, 186, 195, 286]. The period elapsed since the intervention shall also be noted: “Short term” refers to around 1 year, and “long term” to 5 years and longer.

##### Recommendation 87

Assessment of the recurrence rate should distinguish between technical (duplex, reflux finding) and clinical recurrence (visible varicose veins, painful symptoms, quality of life). Clinical recurrence may arise from a technical recurrence.

Recurrences originating in the SFJ and SPJ regions are particularly significant, since as a rule they can lead to a haemodynamically important recurrence of a varicose vein [287].

A retrospective long-term study of 830 operated legs showed a recurrence rate of less than 3% in the junction region on duplex ultrasound up to 20 years after the operation [200]. The technical recurrence rates in the groin region reported in prospective studies with modern operating techniques (reflux on duplex ultrasound) are around 3% to 5% after 5 years [174, 185, 252]. In the largest multicentre study published in Germany, including over 1000 legs (LaVaCro), the recurrence rate in the SFJ (duplex ultrasound) was 2.6% after 2 years [184]. The often higher recurrence rates reported in the literature, associated with the concepts of high ligation, saphenofemoral ligation, and flush ligation, shall be interpreted with care, as the operations thus described are not necessarily the same as the crossectomy procedure described above [288–290]. The clinical recurrence rate is 20%–45% after 5 years [174, 185, 186, 194, 200, 287]; it includes both recurrence in the course of the treated vein and new varicose veins in other regions. This is due not only to the method used but also to the progression of the chronic varicose condition [3, 158, 187, 291–293].

##### Recommendation 88

Varicose vein treatment by open operative procedures offers the possibility of good postoperative outcomes with a clear improvement in quality of life. After technically correct crossectomy of the GSV, very low recurrence rates in the SFJ are reported: less than 5%, even in the medium to long term (5 years to more than 10 years). This should be taken into consideration, since recurrence in the SFJ as a rule also leads to haemodynamically important recurrence of the varicose vein.

**6.2.1.2.5 Prophylactic measures against recurrence**

The following are known to cause recurrence:

- Leaving a stump of GSV, incomplete crossectomy
- Failure to close the reflux source, e.g., reflux from the pelvic region
- Neovascularisation

Modern operating tactics should therefore include the following to prevent recurrence, in addition to correct execution of the crossectomy:

- Exact preoperative mapping with duplex ultrasound
- Use of nonabsorbable ligation material/suture at the SFJ [198–200]
- Techniques to isolate the free endothelium from the GSV stump (coagulation of the free endothelium [294], stitching over and depression of the stump border [196, 295])
- Barrier operations with suture/plastic closure of the cribriform fascia [294, 296] and [297, 298]

##### Recommendation 89

Various measures can be included as prophylactics against recurrence in varicose vein surgery by the open operative procedure. These measures include preoperative mapping by duplex ultrasound, use of nonabsorbable suture for ligation of the SFJ, and techniques for closing the free endothelium of the SFJ stump and for closing the saphenous opening.

##### 6.2.1.3 Surgical strategies that preserve the saphenous vein.

As an alternative to established procedures, which are based on the removal or destruction of the saphenous vein, the reflux can be stopped using strategies and techniques to recover valve function.

A vein-preserving treatment may be indicated, such as when the presence of atherogenic risk factors suggests that arteriosclerotic circulation disturbances may arise in the future, with the GSV possibly being needed as replacement vessel material.

The feasibility of vein-preserving procedures is dependent on certain conditions. In cases of advanced disease with existing damage to the vein valves and sections of wall, for example due to thrombosis, vein-preserving treatment is generally no longer possible.

The following procedures are available in Germany, although they are relatively seldom used:

- Extraluminal valvuloplasty
- CHIVA (Cure Conservatrice et Hémodynamique de l’Insuffisance Veineuse en Ambulatoire, i.e., conservative haemodynamic ambulatory treatment of venous incompetence)

**6.2.1.3.1 Extraluminal valvuloplasty (EVP)**

Introduction

In extraluminal valvuloplasty (EVP), the dilated GSV is reduced to its physiological diameter in the region of the SFJ by placing a restrictive cuff around the outside of the vein. The cuff is made of a piece of alloplastic material measuring 4 cm× 2 cm. The venous valve wings, which did not close due to the stretching of the vein, are thus brought back into contact and can carry out their function of closing the vein [299, 300].

##### Recommendation 90

Extraluminal valvuloplasty can be used as a procedure to preserve the saphenous vein in suitable patients. Its object is to reestablish valve function in the SFJ region. This can lead to an improvement in saphenous vein function.

**6.2.1.3.1.1 Indications**

An essential precondition for EVP is ultrasound proof that the wings of the terminal valve of the GSV are intact and mobile. The vein diameter measured at the height of the terminal valve in the standing patient should not exceed 10 mm in women or 12 mm in men [300].

**6.2.1.3.1.2 Recommendations for execution**

The operative approach is the same as for crossectomy, with additional viewing of the deep vein. The cuff of alloplastic material (polyester, polytetrafluorethylene, or polyurethane) is applied to reduce the diameter of the dilated vein to around 6 mm. The proximal ends of the cuff are fixed to the deep vein. The success of the intervention can be checked intraoperatively with clinical tests or ultrasound. Complementary treatment of the varicose tributary vein can be carried out in the same session or after an interval by various methods.

**6.2.1.3.1.3 Undesired effects**

Postoperative thrombus formation in the GSV occurs in approximately 2% of cases [301–303]. Wound infections occur with the same frequency observed after crossectomy operations. If deep wound infection occurs (frequency < 1%), the alloplastic material shall be removed [300].

**6.2.1.3.1.4 Outcomes**

Long-term recovery of valve function was achieved in 59% to 96% of cases in studies with follow-up periods of 5–10 years. Patients in whom the treatment failed were reoperated with crossectomy and stripping in around 3.7% to 15.6% [300].

**6.2.1.3.2 CHIVA procedure**

The CHIVA procedure is a strategy for treating venous incompetence while preserving the saphenous veins and the draining perforator veins [304].

After analysis of the recirculation with duplex ultrasound, the patient is classified on the basis of the reflux source and the drainage paths, and treatment is planned [305, 306]. After the intervention, the saphenous vein recovers its tone because of the volume relief. In 20% of cases, two sessions are required for the procedure.

**6.2.1.3.2.1 Indication**

##### Recommendation 91

The CHIVA procedure can be used in all clinical stages of primary varicose veins. It is not recommended in cases of postphlebitic alterations to the saphenous vein or reflux in very thin saphenous veins, as calibre reduction is not possible in these cases.

**6.2.1.3.2.2 Recommendations for execution**

The following technical steps are used in the CHIVA strategy, either alone or in combination [305]:

1. Closure of the saphenofemoral and/or saphenopopliteal junctions by ligation [307, 308]. Nonabsorbable sutures shall be used in surgical crossectomy as part of a CHIVA treatment (preserving the saphenous vein);
2. Perforator vein interruption (in the rare cases in which a perforator vein is the highest reflux source);
3. Tributary ligation in combination with 1 and 2;
4. Tributary interruption in isolation to restore circulation in the refluxing saphenous vein. It is carried out through a sufficiently large incision to allow flush ligation of the tributary at the saphenous vein [305, 306];
5. The saphenofemoral and saphenopopliteal junctions and some perforator veins can be closed with endoluminal procedures [43, 309].

**6.2.1.3.2.3 Prophylactic measures against recurrence**

- Exact preoperative mapping
- Nonabsorbable sutures at the junction

**6.2.1.3.2.4 Undesired effects**

General surgical complications are as described above. Superficial thrombosis may occur after the intervention in the preserved saphenous vein (1.3%–8%).

**6.2.1.3.2.5 Outcomes**

The overall recurrence rate detected by duplex ultrasound (reflux in superficial leg veins of over 1 s in duration) is between 18% and 22% after 5–10 years [304, 307, 308].

This breaks down as follows: 2.9% in the SFJ/SPJ, 1.4% from vessels in the pelvic region, and 18.5% reflux from the preserved GSV into distal tributaries without reflux from the deep leg veins, but from competent, more proximal tributaries.

##### 6.2.1.4 Flanking measures and posttreatment.

Preoperative measures may be necessary to decongest oedemas and cure skin alterations caused by congestion or infection. Perioperative antibiotic prophylaxis should not be administered routinely, but it may be necessary in certain cases [310]. Some authors describe the use of blood evacuation during varicose vein surgery to reduce haematomas [139, 262, 276, 311–313]. No definitive assessment of this practice is possible due to the lack of studies to date.

Compression treatment shall be applied with bandages or stockings immediately after the operation. The recommendations of the guidelines on medical compression treatment of the limbs with medical compression stockings (MCS), phlebological compression bandages (PCB), and medical adaptive compression systems (MACs) apply. The patient shall be mobilised immediately.

Thromboembolic drug prophylaxis is not indicated in principle; however, it may be applied depending on the individual risk profile, number of invasive measures carried out, type of general anaesthetic, and duration of the intervention and postoperative immobilisation [276]. The recommendations of the thromboembolic prophylaxis guidelines apply.

Routine postoperative measures include the following:

- Early mobilisation [172]
- Compression treatment for at least 1 week [101, 172, 314, 315]. Longer compression treatment after an operation on an uncomplicated varicose vein is not unanimously supported by the literature
- Wound monitoring, changing of bandages, and removal of stitches if used. Complications such as wound infections, haematomas, or new swellings are recognised and treated in this context
- It is a good idea to check the success of the treatment postoperatively by duplex ultrasound

Further examinations are indicated if new varicose veins appear or if progression of the chronic venous incompetence continues.

##### Recommendation 92

Immediately after varicose vein operations, compression treatment shall be applied for at least 1 week. The patient shall be mobilised immediately.

##### Recommendation 93

Thromboembolic drug prophylaxis is not indicated in principle; however, it may be applied depending on the individual risk profile and the characteristics of the intervention (extent, duration, etc.).

##### Recommendation 94

To ensure the success of the treatment, a postoperative examination should be carried out by duplex ultrasound.

**6.2.1.5 Recurrent varicose veins.**

##### Recommendation 95

Any varicose veins that appear in a previously treated region shall be considered recurrent varicose veins, independent of the type of previous treatment.

Recurrent veins after treatment can be divided into duplex ultrasound recurrence and clinical recurrence. Duplex ultrasound recurrence as a surrogate parameter frequently precedes clinical recurrence [14, 316, 317].

Recurrence is a socioeconomic problem, since up to 20% of all interventions are for cases of recurrence [153, 318–320]. Varicose vein recurrence occurs most frequently in the regions around the saphenofemoral junction and the saphenopopliteal junction and in perforator veins [321]. In many cases, symptoms of varicose vein recurrence first appear some 7–8 years after initial treatment [322]. Multiple pregnancies and high body mass index are considered risk factors for varicose vein recurrence [323, 324]. Pelvic incompetence can also lead to varicose vein recurrence [325].

The following are currently considered to cause varicose vein recurrence [326, 327]:

1. Tactical and technical errors in the initial operation and/or recanalisation or nonclosure in an endovenous procedure
2. Neovascularisation
3. Progression of the underlying disease

These causes are currently the focus of intensive research. There is insufficient evidence for a reliable, evidence-based assessment as to which procedure results in the lowest number of clinical recurrences [174, 185, 186, 328, 329]. Duplex ultrasound detection of recurrence in the junction region 5 years after treatment is significantly less frequent after crossectomy and stripping or endovenous thermal ablation than after ultrasound-guided foam sclerotherapy [330, 331]. Moreover, duplex ultrasound detection of recurrence in the junction region 5 years after treatment is significantly less frequent after crossectomy and stripping than after endovenous thermal ablation [330, 332].

To date, there is no unified definition of varicose vein recurrence in most studies, so the published causes and recurrence rates are frequently not comparable.

##### Recommendation 96

In clinical practice, varicose vein recurrence should be divided into clinically significant recurrence and recurrence diagnosed only by duplex ultrasound.

##### Recommendation 97

Indications for the treatment of recurrent varicose veins should be based on the same principles as those for primary treatment.

Care should be taken because the complication rates of interventions on recurrent varicose veins are higher than for primary interventions.

The whole range of treatments can be considered for recurrent varicose veins. Various barrier techniques are recommended for avoiding postoperative neovascularisation [296, 333–335].

The few published studies that recommend one procedure or another do not satisfy the criteria of evidence-based medicine.

The complication rates for interventions on recurrent varicose veins are higher than for primary interventions [256, 296, 326, 336, 337].

#### 6.2.2 Endovenous procedures

In 1999, Boné reported for the first time the closure of an incompetent saphenous vein using endovenously applied laser light [338]. Radio-frequency treatment of a varicose saphenous vein was described at the same time [339]. Since then, endovenous thermal treatment has developed into a standard treatment method. An integral component of all endovenous procedures is ultrasound before, during, and after treatment [340]. Good knowledge of the ultrasound technique and venous anatomy is necessary.

##### Recommendation 98

To carry out endovenous thermal treatments, the operator shall have very good knowledge and extensive experience in ultrasound representation of the peripheral veins.

##### 6.2.2.1 Endovenous thermal ablation procedures (EVTA).

**6.2.2.1.1 Indications and contraindications**

Definite indications for endovenous thermal ablation (EVTA) procedures are varicose GSV and SSV [340, 341]. Applications in the anterior accessory saphenous vein (AASV) and posterior accessory saphenous vein (PASV), incompetent perforator veins, and venous malformations have been described, with good outcomes [88, 342–344].

In addition to the general contraindications for elective operational interventions in the vein system, acute superficial vein thrombosis of the saphenous vein to be treated is also a contraindication.

##### Recommendation 99

Indications for EVTA should be incompetent GSV, SSV, or anterior accessory saphenous vein (AASV). Incompetence of the posterior accessory saphenous vein (PASV), perforator veins, long varicose segments in cases of venous malformation, and varicose vein recurrence may also be indications.

**6.2.2.1.2 General recommendations for execution of endovenous thermal ablation procedures**

In the endovenous thermal ablation procedure (EVTA), the vein to be treated is either opened or, preferably, punctured, as a rule at the distal reflux point. A treatment probe is introduced into the vein through a special peripheral venous catheter or a lock (Seldinger technique). In thermal ablation of the SSV, choosing a puncture site in the middle of the calf rather than the supramalleolar region is associated with a reduction in postoperative paraesthesia [345].

##### Recommendation 100

To avoid sensory nerve damage, it may be a good idea not to treat the SSV with thermal techniques below the mid-calf.

The intravasal position of the endovenous catheter in the treated vein shall be controlled by ultrasound throughout the length of the vein. The probe tip shall be pushed up to the proximal reflux source close to the junction with the deep vein and positioned under ultrasound control. The final position of the probe tip shall be documented with an ultrasound image. Thermal damage of the deep vein shall be avoided. At the same time, occlusion shall be achieved from close to the junction. Leaving a long stump should be avoided to minimise the risk of recurrence of the reflux [322, 330, 346].

##### Recommendation 101

The correct intravasal position of the endovenous catheter and the position of the probe tip shall be controlled and documented by ultrasound.

Thermal ablation is usually carried out after infusion of a perivenous fluid heat sink (tumescent solution) [347]. To avoid damage to perivenous tissue and to improve contact between the treatment probe and the vein wall, the tumescent solution is applied intrafascially [341, 348–353].

For the legal prerequisites for tumescent local anaesthesia (TLA), see Sect. 6.2.

##### Recommendation 102

Endovenous thermal ablation shall be carried out under the protection of a perivenous fluid heat sink (tumescent solution).

Endovenous thermal ablation is usually carried out in either the horizontal or the Trendelenburg position [354].

Simultaneous treatment of varicose tributaries during EVTA of the GSV is linked to lower frequency of postoperative follow-up interventions, significantly better quality of life, and significantly lower severity of the disease than EVTA alone [355–357].

##### Recommendation 103

If varicose tributaries are present, they should be treated simultaneously with EVTA of the saphenous vein.

Postoperative compression treatment is used wherever possible after EVTA procedures [97], for a period that may vary between 2 days and 6 weeks. Different means of compression may be used. Some prospective randomised studies have shown advantages with respect to painful symptoms, use of analgesics, and quality of life in patients who received compression treatment after laser ablation [93, 99, 358].

##### Recommendation 104

Compression treatment should be applied after endovenous thermal ablation. The duration of compression treatment can be established individually.

For postoperative thrombosis prophylaxis, please see the current S3 guidelines on venous thromboembolism (VTE) prophylaxis [359]. No systematic clinical studies have yet been published on the subject of routine postoperative thrombosis prophylaxis after EVTA procedures.

##### Recommendation 105

After endovenous thermal ablation, venous thromboembolism (VTE) prophylactic drugs should be administered in accordance with the thrombosis prophylaxis guidelines.

One advantage of EVTA is that it can be carried out safely and effectively without interrupting therapeutic anticoagulation [360]. Patients who were receiving anticoagulants presented no more bleeding than patients with no such treatment, as long as no varicose tributaries were removed [361].

##### Recommendation 106

Endovenous thermal ablation can be carried out in patients receiving therapeutic anticoagulation without interrupting anticoagulation.

##### 6.2.2.2 Endovenous laser ablation (EVLA).

In endovenous laser ablation (EVLA), laser generators emitting wavelengths of 810–1940 nm are used. With shorter wavelengths (810–980 nm), most of the laser energy is absorbed by the haemoglobin in the erythrocytes, whereas longer wavelengths (≥ 1320 nm) are absorbed mainly by water.

The probe tip may be a simple glass fibre (bare fibre) or a modified laser-emitting fibre (e.g., radial tip, tulip tip, jacket tip). Bare tips emit the laser beam axially. Tulip tips and jacket tips are used to centre the bare fibre in the lumen of the vein. Radial tips emit laser towards the vein wall in a ring through an optical prism. The laser energy causes thermal damage to the vein wall (photon absorption in the tissue). This results in variably expressed collagen denaturation and shrivelling of the treated vein, and finally thrombotic vein closure [362].

##### Recommendation 107

To optimise treatment outcomes, improvements in the technologies/wavelengths/probe tips should be taken into account in the treatment decision.

**6.2.2.2.1 Special recommendations for execution of EVLA**

Because of the risk of perforation, the sharp bare fibre tip should be introduced and positioned only using a special guide catheter. The pilot beam alone is insufficient for exact positioning of the laser tip [340].

The laser energy is applied during continuous or stepped withdrawal of the laser probe. The energy is emitted in pulsed or continuous laser mode.

##### Recommendation 108

A bare fibre tip shall be introduced and positioned only with a special guide catheter.

##### Recommendation 109

Treatment protocols with continuous withdrawal should be preferred for EVLA. The speed of withdrawal depends on the desired energy density.

##### Recommendation 110

In EVLA, the energy density, power in watts**,** and speed of withdrawal in millimetres per second should be adapted to the laser wavelength used and to the vein lumen.

##### Recommendation 111

The type of application, laser tip, and wavelength used, as well as the tumescent solution composition and volume, shall be documented. This documentation shall include the power in watts, the total energy in joules, the length of vein treated in centimetres, the laser mode, and, if appropriate, the pulse protocol.

The energy density in the treated vein is decisive for the effectiveness of EVLA. Most studies are based on either the linear endovenous energy density (LEED) in J/cm or the endovenous fluence equivalent (EFE) in J/cm^2^, which takes into account the diameter of the treated vein.

##### Recommendation 112

The laser energy density (LEED) applied should be in the range of 60–100 J/cm of vein. The energy density should be adapted to the vein diameter.

The success of EVLA depends on the laser power, the vein diameter, and the withdrawal speed (continuous laser emission) or the pulse duration and interval between pulses (pulse mode). There are indications of a negative correlation between the long-term effectiveness, measured by the occlusion rate or the recanalisation rate, and increasing vein diameter [58, 363].

##### Recommendation 113

When indicating treatment and/or selecting endothermic laser ablation, it may be useful to consider the vein diameter, although a clear threshold value cannot currently be defined.

**6.2.2.2.2 Undesired effects**

Undesired effects are summarised in Table 5.

**Table 5:** Complications and side effects of endovenous thermal treatment of varicose veins

	EVLA 1^a^	EVLA 2^b^	RFA (bRFA)	RSTA (sRFA)	EVSA
**Intraoperative/intraprocedural**					
*Bleeding*	0 (not reported)	0 (not reported)	0 (not reported)	0 (not reported)	0 (not reported)
Requiring transfusion	–	–	–	–	–
*Injury to large blood vessels*	0 (not reported)	0 (not reported)	0 (not reported)	0 (not reported)	0 (not reported)
Arterial	–	–	–	–	–
Venous	–	–	–	–	–
Arteriovenous fistula	Individual cases	–	–	Individual cases	–
*Damage to lymph vessels*	0 (not reported)	0 (not reported)	0 (not reported)	0 (not reported)	0 (not reported)
*Nerve damage*	–	–	–	–	–
Motor nerve	Individual cases	0 (not reported)	0 (not reported)	Individual cases	0 (not reported)
Sensory nerve	0–17	0–9	2–13	2–12	1–10
Other	–	–	0	–	–
*Other neurological reactions/events*	0 (not reported)	0 (not reported)	0 (not reported)	0 (not reported)	0 (not reported)
*Allergic reaction*	0 (not reported)	0 (not reported)	0 (not reported)	0 (not reported)	0 (not reported)
*Burns*	0–3	0–0.5	0	0–1	0
*Probe errors/punctures/injections*	0 (not reported)	0 (not reported)	0 (not reported)	0 (not reported)	0
**Postoperative/postprocedural**					
*Secondary bleeding*	0 (not reported)	0 (not reported)	0 (not reported)	0 (not reported)	0 (not reported)
Requiring revision	–	–	–	–	–
Requiring transfusion	–	–	–	–	–
*Haematoma*	0–10	0 (not reported)	0 (not reported)	0 (not reported)	0 (not reported)
Puncture/surgical haematoma removal	?	–	–	–	–
*(Suffusion) better: Ecchymosis*	10–92	2–64	0.3–3	0–51	3–47
*Infection*	0–3	0–0.8	0	0–6	0
With wound revision	?	–	–	–	–
With (systemic) antibiosis	?	–	–	–	–
*Healing disturbances*	0 (not reported)	0 (not reported)	0 (not reported)	0 (not reported)	0 (not reported)
*(Skin) necrosis*	0 (cf. burns)	0 (cf. burns)	–	0 (cf. burns)	0
*Pigmentation disturbances*	0–43	0–4	2	2–9	5–8
*Pathological scar formation*	0 (not reported)	0 (not reported)	0	0 (not reported)	0
*Lymphatic fistula/lymphatic cyst/lymphoedema*	0–9	0 (not reported)	0	0–3	0
*Superficial thrombophlebitis*	0–22	0–14	0–2	0–14	8–9
*Deep vein thrombosis*	0.6 (including PASTE)	0–2	0	0–3	0
*Lung embolism*	0 (not reported)	0 (not reported)	0 (not reported)	Individual cases	0 (not reported)
*Occurrence of spider veins/matting*	0–13	0 (not reported)	0 (not reported)	0 (not reported)	0 (not reported)
*Compartment syndrome*	0 (not reported)	0 (not reported)	0 (not reported)	0 (not reported)	0 (not reported)
**Deaths**	0 (not reported)	0 (not reported)	0 (not reported)	0 (not reported)	0 (not reported)

*EVLA* endovenous laser ablation, *RFA* radio-frequency ablation, *bRFA* bipolar radio-frequency ablation, *sRFA* segmental radio-frequency ablation, *EVSA* endovenous steam ablation, *PASTE* postablation superficial thrombus extension

^a^EVLA with short wavelengths (810–980 nm) and bare fibre

^b^EVLA with longer wavelengths (1320 nm, 1470 nm, 1500 nm, 1920 nm, or 1940 nm) and modified laser tips

**6.2.2.2.2.1 Endovenous laser ablation (EVLA) with short wavelengths (810–980** **nm) and bare fibre**

Postoperative pain is reported with frequencies between 21% and 75% [286, 364–368] and maximum intensity between 2 and 6 (visual analogue scale [VAS]_0–10_) [93, 99, 365, 368–381], diminishing considerably in the first 7 to 10 days post procedure [369, 371–373, 376, 378, 380]. Significantly more intense postoperative pain after EVLA with short wavelengths (810–980 nm) and bare fibre was reported in comparison with the following:

- Laser emissions with the same wavelength and modified probe (jacket tip) [382]
- Laser with longer wavelengths (1470 nm, 1500 nm) [383, 384]
- Laser with longer wavelengths and modified probe (1470 nm plus radial tip) [385]
- Radio-frequency ablation (bipolar, segmental) [369, 373, 376, 380, 383, 386, 387]
- Steam ablation [375]

Postoperative internal bleeding in the form of usually moderate ecchymosis is reported after EVLA with short wavelengths (810–980 nm) and bare fibre, with an average frequency of 31% (10%–92%) [93, 286, 364, 365, 367–369, 371, 376, 377, 379, 380, 385, 387–390]. The use of longer wavelengths (980 nm as opposed to 810 nm) is associated with less severe postoperative side effects (ecchymosis, pain, phlebitic reactions) [391]. Haematomas occur considerably less frequently after EVLA, at a frequency of 0–10% [372, 373, 392, 393]. In addition to laser probe perforations, vessels may also be punctured during TLA infusion. In comparison with other thermal ablation procedures, it has been shown that a reduction can be achieved in postoperative ecchymosis by the use of laser at longer wavelengths and/or with a modified probe (radial tip, jacket tip) [382, 384, 385] and by the use of radio-frequency procedures and steam ablation [369, 376, 380, 394].

The compression bandaging technique also has an influence on the rate of postoperative ecchymosis and pain; both side effects are significantly reduced by eccentric compression along the course of the GSV [93].

Burns and/or necrosis after EVLA with short wavelengths (810–980 nm) and bare fibre have been described in individual cases (0–2.6%) [286, 364, 365, 368, 369, 375, 377, 379, 384, 385, 387, 390, 393, 395–397].

Sensory nerve damage (paraesthesia, dysaesthesia) has been reported after EVLA with short wavelengths (810–980 nm) and bare fibre, with an average frequency of 2.4% (0–17%) [286, 364–373, 375–378, 384, 385, 387–389, 393–396, 398–404]. The frequency has been decreasing over time [174, 186, 392]. Studies have shown that EVLA in the SSV appears to be associated with a lower rate of sensory nerve damage than in the GSV [372, 405]. A randomised controlled trial (RCT) reported a significantly lower rate of sensory complications with the use of a 1470-nm radial tip compared with a 980-nm bare fibre [385]. No differences in sensory complications have been reported in comparison with radio-frequency ablation (RFA) procedures [369, 373, 376, 387].

Motor nerve damage has been described only in individual cases for thermal ablation of the SSV and/or the vein of Giacomini [406].

Lymphoedemas after EVLA with short wavelengths (810–980 nm) and bare fibre have only been reported by one work group in an RCT, in this case with a 9.2% frequency 2 months after the intervention [366].

Hyperpigmentation after EVLA with short wavelengths (810–980 nm) and bare fibre is reported with frequencies of 0–43%, with an average rate of 31.3% [286, 364, 366–368, 373, 378, 387, 389, 393, 395, 396, 398–400]. Five years after the operation, the frequency drops to 0–4% [186, 392]. No differences have been reported in comparison with RFA procedures [373, 387].

Spider veins as an undesired side effect have only been reported by one work group in an RCT, with a 13.2% frequency 6 months post procedure [286, 364].

Superficial vein thromboses and periphlebitic tissue reactions are reported after EVLA with short wavelengths (810–980 nm) and bare fibre, with an average frequency of 6.5% (0–22%) [93, 365, 368, 369, 371–373, 375–379, 387, 389, 393, 395, 396, 398–401, 403, 404].

No significant differences in the frequency of postoperative phlebitis could be established in comparison with other thermal ablation procedures [386, 389].

##### Recommendation 114

Due to the less than optimal side effects profile of EVLA with short wavelengths (810–980 nm) and bare fibre, a procedure with longer wavelengths and/or a modified probe, or else a new-generation radio-frequency procedure, should be preferred for the treatment of saphenous varicose veins.

Infections after EVLA with short wavelengths (810–980 nm) and bare fibre seldom occur; they may also be associated with phlebectomies carried out simultaneously. Frequencies of 0–3%, with an average of 0.4%, are reported [286, 364, 365, 367–369, 371, 373, 375–379, 384, 389, 392, 393, 398–400]. No differences have been found in comparison with other thermal ablation procedures [386, 389].

Thromboembolic complications can be divided into ablation thrombosis, deep leg vein thrombosis (DVT), and lung embolism.

If a thrombus forms postoperatively at the proximal junction of the treated vein, it is called endovenous heat-induced thrombosis (EHIT) in the international literature; if it extends into the deep vein system, it is called postablation superficial thrombus extension (PASTE) [407–409]. Schäffer et al. indicate that this clinical constellation is independent of the procedure used and can appear even after nonthermal procedures; furthermore, it refers to a thrombus extension in the deep and not the superficial vein system. They therefore suggest the term postablation thrombus extension (PATE) [410].

A Cochrane analysis reported a frequency of thromboembolic events after EVLA with short wavelengths (810–980 nm) and bare fibres of 0.6% (DVT and PATE) [389]. A current meta-analysis, including 16,398 patients treated by endovenous thermal procedures, presents differing frequencies of 1.3% (DVT and PATE), 0.2% (DVT), and 0.1% (lung embolism) after EVLA of the GSV [409].

There are indications from multivariate analyses that a vein diameter > 8 mm (GSV) or > 6 mm (SSV), a previous record of deep vein thrombosis, and male sex are factors that increase the risk of a thrombus extension after thermal ablation [411–414]. The therapeutic procedure depends on the extent of the thrombus extension; however, it is not supported by prospective examinations because of the infrequency of the condition. Different treatment protocols or suggestions are reported in the literature, with or without therapeutic anticoagulation [411–414].

There are various recommendations for graduating the subsequent risks of EHIT and PASTE (classification according to Dexter et al.) [407, 408]. Schäffer et al. suggest the classification presented in Table 6 [410]. The stage-dependent treatment recommendation in each case is based on a consensus of experts.

**Table 6:** Postablation thrombus extension (PATE): classification and treatment recommendations

PATE	Anatomical location	Procedure/treatment
0	Extension of the thrombus as far as the deep vein (= flush closure = desired treatment outcome)	No specific measures required
I	Extension of the thrombus a few millimetres into the deep vein with obstruction of the lumen of the deep vein up to 25%	Duplex ultrasound control (every 1–2 weeks) until regression of the thrombus (level 0)
		Consider anticoagulation in prophylactic dosage
II	Extension into the deep vein with obstruction of the lumen ≤ 50%	Duplex ultrasound control (every 1–2 weeks) until regression of the thrombus (level 0)
III	Constriction of the deep vein > 50% without complete occlusion of the deep vein	Therapeutic anticoagulation until regression of the thrombus to level 0
IV	Complete occlusion of the deep vein	Therapeutic anticoagulation analogous to the treatment of deep leg vein thrombosis
		Regular duplex ultrasound control
		After regression of the thrombus (level 0), suspension of therapeutic anticoagulation can be considered

**6.2.2.2.2.2 Endovenous laser ablation (EVLA) with longer wavelengths (1320–1940** **nm) and modified probes**

In recent years, several clinical examinations have been published in which EVLA was carried out with longer wavelengths (1320–1940 nm) and modified probes. These include special tips to centre the bare fibre in the lumen (tulip tip), a metal sheath (jacket tip), and radial emission probe heads [382, 384, 385, 415–435].

Experimental examinations indicate that EVLA with longer wavelengths (1470 nm) and radial fibres in continuous withdrawal (1–2 mm/s) lead to controllable and reproducible thermal effects on the tissue. Overaccentuated tissue damage such as carbonisation or vein wall perforations, observed with shorter wavelengths and bare fibre [436], occurs less frequently [437]. Due to the greater absorption of laser in water and/or the cytoplasm of the vein wall cells, the use of a longer wavelength allows the same thermal effect on the vein wall to be achieved with lower power or energy density than with shorter wavelengths and higher energy density [384].

A controlled clinical comparative study shows significantly lower undesired effects such as ecchymosis, paraesthesia, and pain in EVLA with longer wavelengths (1470 nm) and a radial probe compared with EVLA with shorter wavelengths (980 nm) and bare fibre [385]. Apart from the use of longer wavelengths, the clinical outcome can also be improved by the use of modified laser emission probes, reducing undesired effects such as ecchymosis, paraesthesia, and pain. This was shown in clinical and experimental examinations with the use of a probe head to centre the bare fibre in the lumen (tulip tip) [427] and a bare fibre surrounded by a metal sheath (jacket tip) [382, 438]. The use of a radial emission probe (radial tip) has also been observed to offer advantages over bare fibre, with reduced ecchymosis and pain and no negative impact on the effectiveness of vein occlusion up to 5 years post procedure [415, 425, 433].

All in all, the risk profile of EVLA with longer wavelengths and a radial tip can be regarded as low. Serious complications are seldom observed (see table with summary of complications/side effects). None of the comparative studies or cohort studies indicated a higher risk of complications or undesired effects with the use of longer wavelengths and modified probes compared with the control groups.

The significance of the laser wavelengths and probes used and their specific influence on the reduction of undesired effects is currently under experimental and clinical investigation [382].

##### Recommendation 115

To reduce undesired side effects such as ecchymosis, postoperative pain, and paraesthesia, modified probes, such as radial emission heads, and longer wavelengths should be preferred.

**6.2.2.2.3 Outcomes**

Since the introduction of endovenous laser ablation 20 years ago, a large number of randomised controlled studies (RCTs) have been carried out. The most extensive data report on first-generation laser systems, particularly short wavelengths (810–980 nm) and bare fibre probes [439]. The majority of RCTs and current case control studies report on the treatment of GSV incompetence, while considerably less information is available about endothermal ablation of the SSV.

There are countless different treatment protocols using different laser generators and/or wavelengths and probes, as well as different power settings, withdrawal speeds, and energy densities.

To date, long-term data from the highest level of evidence (RCTs) according to the Union Internationale de Phlébologie (UIP) definition [46] are available almost exclusively for laser with short wavelengths (810–980 nm) and bare fibre.

**6.2.2.2.3.1 Endovenous laser ablation (EVLA) with short wavelengths (810–980** **nm) and bare fibre probe**

Varicose saphenous veins can be treated effectively by endovenous laser ablation [386, 389, 439–441]. The quality and effectiveness of the treatment can be detected in the short term by the occlusion rate, the side effects spectrum, and the convalescence period; in the medium to the long term, they are shown by their impact on quality of life, postoperative evolution of the disease, and the frequency of recurrence, which may be manifested on duplex ultrasound and/or clinically.

Postoperative quality of life

On completion of the immediate postoperative phase up to a period of 5 years after EVLA, there is a significant improvement in quality of life both in relation to the disease and generally [185, 186, 188, 194, 286, 355, 356, 364, 365, 367–369, 371–376, 378, 392, 398–400, 405, 442, 443]. No significant differences have been found in the postoperative quality of life as compared with other thermal ablation procedures [386, 389, 441].

Postoperative evolution of disease severity

A significant improvement in disease severity has been shown for up to 5 years after EVLA [185, 186, 194, 286, 355, 356, 364, 365, 368, 371–373, 375, 376, 378, 385, 387, 392, 400, 401, 405, 442, 443]. Prospective studies, meta-analyses, and systematic reviews show similar values for EVLA with short wavelengths (810–980 nm) and bare fibre as compared with segmental radio-frequency thermal ablation (sRFA) and endovenous steam ablation (EVSA) [375, 386, 389, 441, 444]. Both EVLA with 1470 nm and a radial probe [385] and sRFA [376] were shown to be superior to EVLA with short wavelengths (810–980 nm) and bare fibre, although only up to 1 month after the interventions.

Convalescence

In a comparison of different treatment methods for GSV ablation, EVLA with short wavelengths (810–980 nm) and bare fibre was shown to be inferior to EVLA with a radial probe [385] and to EVSA [375], but it was similar to radio-frequency ablation (sRFA, bipolar radio-frequency ablation [bRFA]) [369, 373, 387].

Technical–anatomical success (duplex ultrasound)

Studies consider the treatment to be immediately effective, with provable occlusion rates of 95% to 100% on duplex ultrasound [355, 365, 368, 369, 372, 374, 376, 377, 380, 385, 393–396, 400, 401, 403]. After 4–5 years, between 85% and 88% of the treated saphenous veins are still occluded [330, 440]. Compared with other thermal ablation procedures (EVLA with longer wavelengths, sRFA, bRFA, EVSA), EVLA with short wavelengths (810–980 nm) and bare fibre appears to present similar anatomical treatment success rates [369, 375, 380, 384, 385].

There are indications in the literature that the risk of recurrence after EVLA with short wavelengths (810–980 nm) and bare fibre and/or reopening of the ablated vein with progressive vein symptoms (C3 and C4 vs. C2) [445] correlates with the vein diameter [393, 396, 397, 401, 403]; different studies give different critical values for maximum GSV diameter of 8 mm, 9 mm, or 12 mm [393, 396, 401]. In another study, no such correlation could be shown [404]. A further factor affecting the occlusion rate with EVLA is the energy density, which with the use of LEED (linear endovenous energy density) should be at least 60 J/cm [393, 404, 446].

Clinical recurrence/progression of the disease

Clinical recurrence, in the sense of all new postoperative varicose veins, regardless of their location and origin, occurs after EVLA of the GSV with short wavelengths (810–980 nm) and bare fibre in 4% of cases after 1 year, in 16% to 26% of cases after 2 years, and in 45% to 47% of cases after 5 years [185, 186, 368, 400, 443]. Clinical recurrence with origin in the region of the previously operated veins, particularly the SFJ region, is reported at frequencies between 8% and 33% at 5 years after EVLA of the GSV with short wavelengths (810–980 nm) and bare fibre [174, 186, 392]. Due to the use of nonunified study variables, no assertions can be made at present about the long-term effectiveness against clinical recurrence in comparison with other treatment procedures.

**6.2.2.2.3.2 Endovenous laser ablation (EVLA) with longer wavelengths (1320–1940** **nm) and modified probes**

To assess the outcomes of EVLA with long wavelengths (1320–1940 nm) and modified probes, randomised controlled studies [384, 385, 416, 417, 420, 426, 427, 430, 431], prospective comparative studies [415, 418, 419, 422, 425], retrospective comparative studies [429, 433], and prospective cohort studies [382, 421, 424, 428, 434] are available. The long-term outcomes up to 5 years are reported.

The long-term effectiveness of EVLA with longer wavelengths and radial probes can also be deemed to be high. A prospective comparative study with the longest observation period reported to date documented an occlusion rate of 96.7% after 5 years [415]. If the results of all the available studies are taken together, they show occlusion rates of between 87.5% and 100% after observation periods of 3 months to 5 years.

The pain levels and requirement for analgesics are low in all clinical studies of EVLA with longer wavelengths and modified probes. The convalescence is short. The average interval from the operation to resumption of normal physical activity is reported as between 0 and 2 days.

##### 6.2.2.3 Endovenous radio-frequency ablation (RFA).

Two types of radio-frequency treatment are currently authorised in Germany. One is segmental thermal ablation using radio frequency (sRFA) at 120 °C; the other, called radio frequency–induced thermal therapy or bipolar radio-frequency ablation (bRFA), delivers the heat directly to the vein wall through a bipolar probe head, heating it to between 60 °C and 100 °C [447].

In RFA in general, the thermal energy causes homogeneous damage to all the layers of the vein wall, with contraction of the collagen fibres in the vein wall resulting in occlusion of the vein. Homogeneous thermal damage of the vein wall was shown ex vivo in a cow’s foot model [436], and occlusion of the vein by fibrosis in an animal experiment [448]. Postablation fibrosis of the vein wall by bRFA has also been shown in vivo and in an ex vivo experiment [449, 450].

**6.2.2.3.1 Special recommendations for execution (method and technique)**

In sRFA, the probe head (7 cm or 3 cm) is heated to 120 °C by radio-frequency energy. The energy produced by the generator is delivered to the probe head by a reverse-coupling mechanism, keeping it continuously at 120 °C. Thermal ablation shall be carried out twice in the junction segment and once in the remaining vein segments.

Contact between the treatment probe and the vein wall can be improved by external compression and positioning [354].

##### Recommendation 116

Radio-frequency ablation can be carried out in the Trendelenburg position. Contact between the treatment probe and the vein wall can be improved by external compression to optimise treatment results.

**6.2.2.3.2 Undesired effects**

Undesired effects are summarised in Table 5.

**6.2.2.3.2.1 Radio frequency–induced segmental thermal ablation/segmental radio-frequency ablation (sRFA)**

The following complications and undesired effects have been described:

- Nerve damage/paraesthesia [436, 451]
- Ecchymosis [376, 451, 452]
- Haematomas [451]
- Induration of the saphenous vein [349, 376, 452]
- Pigmentation [376]
- Phlebitis of the saphenous vein [451, 452]
- PATE [409, 410, 412, 453], c.f. Sect. 6.2.2.2.2.1 PATE after EVLA
- Burns [454]
- Deep leg vein thrombosis [409, 454, 455]
- Lung embolism [409, 455]
- In individual cases: lymphocele, wound infection, arteriovenous fistula [349, 386]

The incidence of paraesthesia after sRFA in the European cohort study was 3.4% in the first postoperative week, and then it fell to 0.4% and remained at that value throughout the 5‑year observation period [451, 456]. This represents a clear improvement over previous treatment procedures [351].

Ecchymosis, generally caused by the application of tumescence, is more frequently described in the perioperative context, with a reported incidence between 5.8% [451] and 33.3% [376].

Induration and shortening of the treated saphenous vein arise due to thermal alteration. Thin patients feel this process, and the thickened vein can be felt under the skin. After around 4–6 weeks it regresses, and the vein can no longer be felt. There are no robust figures in the literature on this side effect and its regression.

Pigmentation as an undesired effect is described in the European multicentre study as occurring perioperatively in 2.4% of cases [451], with persistence after 5 years in 0.4% [456].

The incidence of accompanying superficial vein thrombosis (phlebitis) was 1.0% in the European multicentre study [451] and 8.2% in a randomised multicentre study [444].

Skin burns are rare (0–1.3%) [369, 456]. They occur less frequently with the consistent application of tumescent solution, which was not always done when the method was first developed [457].

Thromboembolic complications occur with a frequency of 0–3.4% [373, 456, 458, 459]. The current meta-analysis cited above showed differing frequencies of 1.4% (DVT and PATE), 0.5% (DVT), and 0.1% (lung embolism) after RFA of the GSV [409].

The reported incidence of PATE after sRFA was 3% in SSV treatment [412] and 1.2% to 2.4% after GSV ablation [409, 453]. More recent works have shown that there is a higher risk of PATE when tributary extirpation is carried out in addition to RFA and when the patient has a prior history of DVT [460]. In addition, vein diameter > 10 mm and intervention time > 40 min are described as independent risk factors for the occurrence of a PATE [461]. For treatment of a PATE, see Table 6.

**6.2.2.3.2.2 Radio frequency–induced thermal treatment/bipolar radio frequency ablation (bRFA)**

There is little literature on bRFA. Ten articles have been published since 2007, including one randomised controlled study [380], one prospective nonrandomised study [387], four prospective cohort studies [447, 450, 462, 463], two prospective studies [464, 465], one retrospective study [466], and one review [354]. The observation periods range between 3 months and 12 months; no long-term studies have yet been published.

Postoperative pain after bRFA is reported with frequencies of none to 74%, with an intensity of 0–2 on the visual analogue scale (VAS) of 0–10 [380, 387, 447, 462, 463].

Postoperative internal bleeding is generally rare, reported with a frequency of 0.9–3% [380, 387, 463, 465].

Burns are not described.

Sensory nerve damage (paraesthesia, dysaesthesia) is reported as the predominant side effect by several authors, with incidence of 1.7%–12.5%; this shows a statistically significant tendency to occur after treatment of the SSV [387, 462, 463].

There are no reports in the literature to date of motor nerve damage, lymphoedemas, or spider veins after bRFA treatment.

Postoperative hyperpigmentation occurs fairly rarely, in 0–1.5% [380, 387, 463, 465].

Superficial vein thrombosis (thrombophlebitic symptoms) is described by only one work group, with a frequency of 2.4% [463].

Serious complications such as deep leg vein thrombosis or lung-artery embolism are not described in the current literature.

**6.2.2.3.3 Outcomes**

Technical success (feasibility)

With proper patient selection (preoperative duplex ultrasound of the course of the saphenous vein), probing and thermal treatment of the saphenous vein is possible in over 95% of patients [451].

**6.2.2.3.2.1** **Radio frequency–induced segmental thermal thermal ablation/segmental radio frequency ablation (sRFA)**

The data availability for outcomes after treatment of superficial vein incompetence is good. There are numerous retrospective analyses. The data from a prospective multicentre cohort study were published up to 5 years post procedure [456], and there are several prospective randomised controlled studies of sRFA vs. EVLA [369, 373, 376] and of sRFA vs. crossectomy/stripping vs. EVLA vs. foam sclerotherapy [331, 444], as well as comparative studies on improvements in quality of life and venous symptoms. There are also meta-analyses comparing the different treatment procedures with one another [386, 389].

The rate of perioperative painful symptoms is very low and sometimes better than the rate for EVLA [376, 444]. As a rule, the resumption of everyday activities occurs sooner than after EVLA [444].

In a Danish prospective randomised multicentre study, the rate of reflux in the GSV 1 year after sRFA was found to be 4.8%. The rate for EVLA was 5.8% [444]. The outcomes were constant over 5 years. The occlusion rate in the GSV 5 years after the sRFA intervention was 94.2% [331].

The occlusion rate in a prospective clinical multicentre cohort study 60 months post procedure was 91.9 ± 1.8%. A significant improvement in the VCSS over the whole study period of 60 months was also shown in the European cohort study, as well as a permanent improvement in the C state [456].

Some prospective randomised studies show significant improvement in quality of life after sRFA up to 60 months post procedure [376, 415, 467].

**6.2.2.3.2.2** **Radio frequency–induced thermal treatment/bipolar radio frequency ablation (bRFA)**

Because of the short observation periods, the published studies of bRFA can make only limited assertions on postoperative quality of life and the postoperative evolution of the severity of the disease after treatment by bRFA. One work group reports an improvement in the VCSS of 79.3% on the original values after 12 months and a highly significant improvement in the digital photoplethysmography values after 3 months and 12 months [387].

In this study, convalescence and return to normal activity after bRFA is given as being within 3 days for 92.4% of patients.

Technical–anatomical success (duplex ultrasound)

Six studies of bRFA have been published since 2009, showing occlusion rates of 74%–98% after 3–12 months [380, 387, 447, 462, 463, 465]; however, they are comparable only up to a point because different treatment protocols were used. The power applied ranged from 18 W to 26 W and the impedance-guided withdrawal speed from 0.5 cm/s to 2 cm/s. A slower withdrawal speed is associated with a higher occlusion rate, as it results in higher energy delivered. The majority of authors recommend power of 18 W and withdrawal speed of 0.5–0.7 cm/s [380, 387, 462, 463, 465].

The execution varies considerably in individual studies, so no clear recommendations can be given [387, 462, 463].

##### 6.2.2.4 Endovenous steam ablation (EVSA) and other procedures. 6.2.2.4.1 Indication

The steam ablation treatment method (also known as steam vein sclerosis [SVS^TM^] or endovenous steam ablation [EVSA]) was authorised in Germany in 2009. It is indicated for the treatment of GSV/SSV incompetence and large lumen varicose tributaries.

**6.2.2.4.2 Recommendations for execution**

Steam is generated from a few microlitres of sterile water under high pressure (over 200 bar) and is injected into the vein through the catheter tip at a temperature of approximately 120 °C. Sixty joules of heat energy are generated with every burst of steam, spread through up to 10 cm of the treated vein.

**6.2.2.4.3 Undesired effects**

Undesired effects include nerve lesion/paraesthesia in 0.9%–9.6% [375, 468], ecchymosis/haematoma in 3.1%–47.3% [375, 469], phlebitis distal to the treated segment in 8.5% [375], pain in the treated vein after the control exploration in 35% [470], and hyperpigmentation in 4.6%–7.6% [375, 468].

**6.2.2.4.4 Outcomes**

Clinical outcomes of steam ablation have been published in three small prospective case series [469–471], one nonrandomised study in comparison with crossectomy and stripping [468], and one randomised controlled study in comparison with 980-nm laser ablation [375]. The recanalisation rate after 6–12 months post intervention is between 4% and 10% [375, 468–471].

##### 6.2.2.5 Other endovenous thermal procedures.

Endovenous microwave ablation (EMA) of varicose veins as an alternative endothermal treatment method has so far been investigated only in two studies [394, 472]. In the randomised study by Yang comparing it with the stripping operation, 10% of the patients presented skin burns after EMA (*p* < 0.01) [472].

##### Recommendation 117

Because of the lack of data and the high number of skin burns, microwave ablation of varicose veins cannot currently be recommended outside clinical studies.

##### 6.2.2.6 Reflux recurrence from the saphenofemoral region.

From previous studies of varicose vein recurrence after crossectomy and stripping (C/S), we know that leaving a long stump of the GSV and/or the SSV can be a source of recurrence of the varicose vein, sometimes through the AASV [302, 473–475]. With endovenous thermal treatments, the ablation generally ends a few millimetres to a few centimetres below the junction of the GSV or SSV with the deep vein system. As a result, a stump of vein is left behind. Preexisting reflux in the stump can vanish, persist, or reappear at a later date.

We know from studies with an observation period of 5 years or more that reflux in the saphenous vein stump occurs more often after endovenous thermal treatment than after C/S [174, 186, 330, 476]. Most of the data refer to EVLA treatments, while this issue has been investigated only occasionally after RFA or cyanoacrylate treatment [477].

The clinical consequences of this reflux are controversial. In the meta-analysis of Hamann et al., the clinical outcomes after C/S and EVLA are comparable, despite frequent saphenofemoral reflux after EVLA [330]. In the work by Rass and Flessenkämper, reflux in the junction region was more common after EVLA than after crossectomy and stripping; however, clinical recurrence of varicose veins in the treated leg was equally frequent overall [186, 476]. Clinical recurrence originating in the treated junction is more common after EVLA with short wavelengths (810–980 nm) and bare fibre. The significance of this finding cannot yet be judged.

There are few long-term outcomes from comparative studies for the new methods with longer wavelengths and radial emission probes [415, 478, 479], and reflux in the SFJ/SPJ or AASV was not always an endpoint in these studies. Investigation of recurrence from the SFJ/SPJ shall await further studies, particularly RCTs and/or large case control studies.

##### 6.2.2.7 General overview of endovenous thermal ablation procedures (EVTA).

Endovenous laser ablation is a safe, established treatment procedure for the treatment of saphenous vein incompetence of the GSV and SSV; the intrafascial course of the accessory veins of the GSV, such as the AASV and PASV; and the femoropopliteal vein. In technical terms, it appears to remain open to further development. Endovenous laser ablation with first-generation laser using short wavelengths (810–980 nm) and bare fibre has proved to have a worse side effects profile than lasers with longer wavelengths (1320–1940 nm) and modified probes, and also RFA [376, 380]. Compared with crossectomy and stripping, duplex ultrasound recurrence of inguinal reflux and clinical recurrence in general from the junction regions are evidence of poorer long-term effectiveness (studies with observation period of 5 years). Nevertheless, the overall clinical recurrence rate, the improvement in quality of life, and the reduction in symptoms after crossectomy and stripping do not differ significantly from the outcomes after EVLA and RFA [186, 415, 476, 480].

Endovenous laser ablation with laser at longer wavelengths (1320–1940 nm) and modified probes is the current state of the art. Studies show minor side effects and quick postoperative convalescence, as well as good effectiveness with high occlusion rates. However, valid long-term studies and controlled comparative studies with open operation are still lacking for these new procedures. Studies are also lacking that would allow assertions to be made, based on stratification, about individualised indications justified by prognosis of presumably significant parameters (e.g., vein diameter, CEAP classification).

The radio frequency (RFA) treatments currently available, i.e. segmental thermal ablation using radio frequency (segmental radio frequency ablation [sRFA]) and radio frequency-induced thermal therapy (bipolar radio frequency ablation [bRFA]), have also become established as safe and effective treatment procedures for eliminating intrafascial venous reflux. They offer perioperative advantages in terms of pain, and quick resumption of everyday activities. Because of lack of data for bRFA, only limited assertions can be made about the medium and long-term outcomes.

Endovenous laser ablation and sRFA have shown significant improvements in quality of life and the clinical severity (VCSS) of CVI in clinical multicentre comparative randomised studies and cohort studies.

##### Recommendation 118

Endovenous laser ablation and radio frequency ablation with modern procedures to eliminate epifascial reflux in cases of saphenous vein incompetence should be offered to varicose vein patients as alternatives to other treatment procedures.

#### 6.2.3 Chemical procedures

Procedures for vein occlusion by other means than heat include sclerotherapy procedures and nonthermal catheter procedures, particularly mechanochemical endovenous ablation (MOCA^TM^) and vein gluing.

##### 6.2.3.1 Sclerotherapy.

Sclerotherapy refers to tactical elimination of a vein segment by targeted injection of a sclerosing agent. In Germany, polidocanol is authorised as a premixed sclerosing agent for varicose vein elimination. Common salt solution is not authorised for use as a sclerosing agent, and there are no scientific data on its use.

**6.2.3.1.1 Indications**

##### Recommendation 119

Sclerotherapy can be used for all forms of varicose veins.

Sclerotherapy is suitable for all forms of varicose veins, including veins of very varying diameters. Periulcerous varices, genital varicose veins, and spider veins are all clear indications for sclerotherapy. Vascular malformations and seroma after varicose vein surgery can be treated effectively with sclerotherapy. Literature overview: saphenous veins (GSV and SSV) [406, 444, 481–486], tributaries [486–488], perforators [487, 489, 490], reticular varices and spider veins [491–494], remaining and recurrent varicose veins after previous interventions [494–499], pudendal or pelvic varicose veins [494, 500, 501], peri-ulcer varicose veins [502–505], and varicose veins in venous malformations [506–508].

**6.2.3.1.2 Contraindications**

There are few absolute contraindications for sclerotherapy [509–513].

##### Recommendation 120

The absolute and relative contraindications for sclerotherapy shall be observed.

##### Recommendation 121

Absolute contraindications are known allergies to the sclerosant, acute venous thromboembolism, and local infection in the region to be treated or a severe generalised infection.

##### Recommendation 122

For foam sclerotherapy, known symptomatic right-to-left shunt is also an absolute contraindication.

##### Recommendation 123

In patients with known thrombophilia and a high risk for thrombus formation, sclerotherapy should be carried out under additional prophylactic drugs to guard against thrombosis.

**6.2.3.1.2.1 Absolute contraindications**

- Known allergy to the sclerosing agent
- Acute venous thromboembolism
- Local infection in the region of the sclerotherapy or severe generalised infection

For foam sclerotherapy:

- Known symptomatic right-to-left shunt (e.g., symptomatic patent foramen ovale)

**6.2.3.1.2.1** **Relative contraindications (individual risk–benefit assessment is obligatory)**

- Pregnancy
- Lactation (if the indication is urgent, interrupt lactation for 2–3 days)
- Severe peripheral arterial occlusive disease
- Poor general state of health
- High risk for thromboembolism (e.g., known history of thromboembolic events, known severe thrombophilia, active cancer)
- Long-term immobility or bed- patient

For foam sclerotherapy:

- Neurological disorders, including migraine, after previous foam sclerotherapy

In cases of relative contraindications, sclerotherapy (especially foam sclerotherapy) can be carried out—if strongly indicated—under special safety considerations.

**6.2.3.1.3 Execution**

##### Recommendation 124

Sclerotherapy of spider veins and reticular varices (C1) should be applied with the limbs in a horizontal position and using a low-friction syringe.

##### Recommendation 125

The sclerosing agent should be applied with the patient lying down.

##### Recommendation 126

Sclerotherapy of varicose tributaries that are not visible percutaneously, or incompetent saphenous veins or perforator veins, should be carried out under ultrasound control (B mode).

##### Recommendation 127

Direct puncture of a perforator vein should be avoided.

The basis of sclerotherapy is the intravenous application of a sclerosing agent. Sclerotherapy is usually planned and applied in sequence from proximal to distal reflux sources and from larger to smaller varicose veins.

Polidocanol (Lauromacrogol 400) is the only sclerosing agent authorised in Germany. It is available in solutions at concentrations of 0.25%, 0.5%, 1%, 2%, or 3%. The concentration and the dosage of the sclerosing agent are selected in accordance with the calibre of the varicose vein to be treated (see Tables 7 and 8). A maximum dose of 2 mg polidocanol/kg body weight should not be exceeded in any treatment session (e.g., 140 mg polidocanol for 70 kg body weight).

**6.2.3.1.3.1 Liquid sclerotherapy**

##### Recommendation 128

The following recommendations on concentrations and amounts per injection for liquid sclerotherapy should be observed (Tables 7 and 8). Concentrations and amounts are reference values and may be adapted according to the therapist’s assessment.

**Table 7:** Recommended amounts per injection for polidocanol in *liquid* sclerotherapy with single injections [514]

Indication	Volume/injection point
Spider veins (C1)	Up to 0.2 ml
Reticular varices (C1)	Up to 0.5 ml
Varicose veins (C2)	Up to 2.0 ml

**Table 8:** Recommended concentrations in liquid sclerotherapy with polidocanol [514]

Indication	Concentration (%)
Spider veins	0.25–1.0
Reticular varices	0.5–1.0
Small varicose veins	1.0
Medium-sized varicose veins	2.0–3.0
Large varicose veins	3.0

**6.2.3.1.3.2 Sclerotherapy with foam sclerosant**

Numerous studies have shown that foam sclerosants are safe and effective. Foam sclerotherapy can therefore be regarded as an established procedure for the treatment of varicose veins. The application of sclerosant as a foam (polidocanol with ambient air) has been authorised by the German Federal Institute for Drugs and Medical Devices (BfArM) since 2009 [514].

**6.2.3.1.3.2.1 Foam production**

##### Recommendation 129

For all indications, a three-way stopcock (Tessari method) or a two-way connector (double-syringe method), or a similar appropriate method, should be used for production of the sclerotherapy foam.

##### Recommendation 130

For all indications, the foam should be prepared by mixing with ambient air; alternatively, O_2_/CO_2_ can be used.

Detergent-type sclerosants, such as polidocanol, can be converted into a fine-bubble foam by special techniques. Ambient air or other gases (CO_2_) can be used for this purpose.

In Tessari’s method, the foam is produced by mixing liquid and air in two syringes, which are connected by a three-way stopcock. The best mixture ratio of sclerosant to air is between 1:3 and 1:4 [515, 516]. In the double-syringe system, polidocanol is mixed with air by turbulent mixing in two syringes connected by a special two-way connector [515, 516].

**6.2.3.1.3.2.2 Recommendations for execution of foam sclerotherapy**

##### Recommendation 131

In routine foam sclerotherapy, no more than 10 ml of foam should be injected per day/session. Larger volumes of foam may be injected after individual risk–benefit assessment.

##### Recommendation 132

The following concentrations should be observed in proportion to the diameter of the treated vein segment. The suggested concentrations and amounts are reference values and may be adapted according to the therapist’s assessment.

In view of its effectiveness, relatively low volumes of sclerosant are recommended in comparison with liquid sclerotherapy (see Table 9; [444, 486, 488, 490–494, 496–498, 502–508, 512, 517–523]). The foam sclerosant can be applied to the vein to be treated through a cannula or a short or long catheter.

**Table 9:** Recommended concentration of foam sclerosants

Indication	Polidocanol concentration (%)
*Spider veins*	Up to 0.5
*Reticular varices*	Up to 1.0
*Varicose tributary veins*	Up to 2.0
*Great saphenous vein, small saphenous vein*	
< 4 mm	1.0
≥ 4 to ≤ 8 mm	1.0–3.0
> 8 mm	3.0
*Incompetent perforator veins*	1.0–3.0
*Recurrent varicose veins*	1.0–3.0
*Venous malformations*	1.0–3.0

The following additional safety measures apply to foam sclerotherapy: Use a maximum of 10 ml foam, avoid Valsalva manoeuvres, and have the patient remain lying down for a few minutes after application.

**6.2.3.1.4 Outcomes**

##### Recommendation 133

With spider veins and reticular varices (C1), the success of treatment can be assessed from the clinical outcome in the control after sclerotherapy. With varicose veins (C2) and venous malformations, clinical and ultrasound examinations shall be carried out.

The success of sclerotherapy in spider veins, reticular varices, and visible tributaries can be controlled by clinical examination. For other indications, success of the treatment should be controlled by duplex ultrasound. This will allow the clinician to establish whether the treated vein is occluded completely, partially, or not at all. If partial occlusion has occurred, the examination will show whether the segment in question presents antegrade, retrograde, or no flow.

**6.2.3.1.5 Complications and side effects**

If correctly carried out, sclerotherapy is a safe treatment with few side effects. Undesired effects or complications seldom occur [521, 524]. However, undesired effects may be observed (see Tables 10 and 11).

**Table 10:** Serious complications after sclerotherapy

Side effect	Liquid sclerotherapy	Foam sclerotherapy
Allergic reactions/anaphylaxis	Individual cases < 0.01%	Individual cases < 0.01%
Skin necrosis	Individual cases < 0.01%	Individual cases < 0.01%
Neurological reactions/stroke/TIA	Individual cases < 0.01%	Individual cases < 0.01%
Distal deep thrombosis	Rare ≥ 0.01 to < 0.1%	Occasional ≥ 0.1 to < 1%
Proximal deep thrombosis	Very rare < 0.01%	Very rare < 0.01%
Lung embolism	Individual cases < 0.01%	Individual cases < 0.01%
Damage to motor nerves	Individual cases < 0.01%	Individual cases < 0.01%

**Table 11:** Minor side effects after sclerotherapy

Side effect	Liquid sclerotherapy	Foam sclerotherapy
Vision disorders	Very rare < 0.01%	Occasional ≥ 0.1 to < 1%
Migraine, headache	Very rare < 0.01%	Occasional ≥ 0.1 to < 1%
Sensory nerve damage	Individual cases < 0.01%	Rare ≥ 0.01–< 0.1%
Dry cough	Rare ≥ 0.01–< 0.1%	Very rare < 0.01%
Superficial phlebitis	Unclear	Unclear
Matting	Frequent ≥ 1–< 10%	Frequent ≥ 1–< 10%
Hyperpigmentation	Frequent ≥ 1 to < 10%	Frequent ≥ 1 to < 10%
Small areas of skin necrosis	Rare ≥ 0.01–< 0.1%	Very rare < 0.01%

##### Recommendation 134

Attention shall be paid to the side effects of sclerotherapy shown in Tables 10 and 11.

**6.2.3.1.6 Mechanochemical endovenous ablation (MOCA)**

##### Recommendation 135

Mechanochemical endovenous ablation (MOCA) can be used as an alternative to other sclerotherapy methods for saphenous vein sclerotherapy.

The principle of MOCA is based on a combination of mechanical and chemical alteration of the endothelium [525–527]. The rotating wire at the point of the catheter provokes vasoconstriction of the vein.

**6.2.3.1.6.1 Indications**

Based on study data, the indications for the procedure to treat incompetent GSV or SSV of diameter up to 12 mm (level A) [528–530] are the same as for treatment using other catheter-supported or operative procedures [531–533]. Mechanochemical endovenous ablation can be combined with other surgical and endovenous procedures.

**6.2.3.1.6.2 Limitations and contraindications**

The impossibility of probing the vein to be treated is a limitation for the procedure, and a vein diameter > 12 mm is a relative contraindication.

Because of the use of a sclerosant, the procedure is subject to the same relative and absolute contraindications that are applicable to sclerotherapy [534–540].

**6.2.3.1.6.3 Recommendations for execution**

The treatment catheter is introduced into the vein and positioned 2 cm below the saphenofemoral or saphenopopliteal junction (according to the manufacturer’s instructions) under ultrasound control. Depending on the vein diameter, liquid polidocanol at 1%–3% [528–530] can be injected up to the maximum dose of 2 mg/kg body weight per day [514]. The target vein can be probed both antegrade and retrograde [533, 541].

**6.2.3.1.6.4 Outcomes**

Studies published to date report occlusion rates for the GSV and VSP after 6 months and 12 months of 96.7 and 94% [529, 542–544], respectively, with a complication rate described as low and little perioperative pain [545, 546]. The 24-month data for the GSV showed an obliteration rate of 94%–95% with significant improvement in vein-related quality of life [547, 548]. The published data refer to vein diameters of less than 12 mm.

##### 6.2.3.2 Cyanoacrylate.

The vein glue used in Germany is N‑butyl 2-cyanoacrylate.

Only one cyanoacrylate treatment is authorised for varicose vein treatment.

##### Recommendation 136

Saphenous varicose veins can alternatively be treated with cyanoacrylate glue.

**6.2.3.2.1 Indication**

The use of a vein gluing procedure, like other catheter-supported procedures, is indicated for treating saphenous vein incompetence.

**6.2.3.2.2 Recommendations for execution**

The vein is accessed by puncture under ultrasound control (B mode). In the vein gluing procedure, the vein is closed (i.e., glued) by polymerisation after intravasal application of the glue. Use of the vein gluing procedure allows treatment without tumescent local anaesthesia or general anaesthetic. If the glue catheter is pushed forward too close to the deep vein, the glue can enter the deep vein system, leading to severe complications.

**6.2.3.2.3 Outcomes**

The first studies showed saphenous vein occlusion rates of 99% after 3 months [370, 549] and 97.2% or 93% after 1 year [550, 551]. The first long-term outcomes show an occlusion rate of 95.3% after 24 months and 94.4% after 36 months [552, 553].

**6.2.3.2.4 Limitations and undesired effects**

In the cyanoacrylate procedure, the vein diameter appears to lead to limitations: Diameters in excess of 8–10 mm present high levels of recanalisation [554]. After treatment of the GSV, phlebitic reactions occur with a frequency of approximately 11%–20%, appearing a few days postoperatively and persisting for up to 1 month [550, 551]. This reaction has not been reported to date in the SSV.

No definite recommendation can yet be given because of the lack of data.

## 7 Varicose veins in pregnancy

Pregnancy has an impact on the vein system of the lower limbs because of both functional alterations and the structural and hormonal changes that occur. Up to 40% of all pregnant women present a new or progressing varicose vein [357].

### Recommendation 137

Any varicose vein that appears during pregnancy shall be diagnosed by a vein specialist.

Various univariate studies, but not multivariate analyses, have shown that pregnancy is an independent significant risk factor for the appearance or progression of a varicose vein [555, 556].

Women with a history of three or more pregnancies frequently present a varicose vein [557–559].

According to the available data, pregnancy is one of the many risk factors for recurrence of reflux after high ligation and stripping of the saphenous vein. The probability of recurrence in women who become pregnant after varicose vein treatment is increased by a factor of 2.69 compared with women who do not become pregnant [558].

The subjective risk evaluation for the development of venous incompetence [560] and the perception of painful symptoms is variable and age dependent [561].

### 7.1 Treatment

#### Recommendation 138

Patients with varicose veins in pregnancy should be informed about the physiological changes and their course, as well as the risks.

The prophylactic effect of compression treatment to prevent a varicose vein during pregnancy has not been proved [357], but compression can improve the symptoms [357].

No study data are available on operative or interventional measures to treat a varicose vein in pregnancy, since pregnancy is established as an exclusion criterion in all studies.

The use of polidocanol for liquid or foam sclerotherapy in pregnancy has not been shown to be safe by study data from trials in humans [514].

#### Recommendation 139

Invasive treatment of varicose veins during pregnancy should be indicated only in exceptional cases.

## Declarations

For this article no studies with human participants or animals were performed by any of the authors. All studies mentioned were in accordance with the ethical standards indicated in each case.

The supplement containing this article is not sponsored by industry.
